# Modified Peptide Inhibitors of the Keap1–Nrf2 Protein–Protein Interaction Incorporating Unnatural Amino Acids

**DOI:** 10.1002/cbic.201800170

**Published:** 2018-07-18

**Authors:** Nikolaos D. Georgakopoulos, Sandeep K. Talapatra, Jemma Gatliff, Frank Kozielski, Geoff Wells

**Affiliations:** ^1^ UCL School of Pharmacy University College London 29/39 Brunswick Square London WC1N 1AX UK

**Keywords:** Keap1, Nrf2, peptides, protein–protein interactions, unnatural amino acids

## Abstract

Noncovalent inhibitors of the Keap1–Nrf2 protein–protein interaction (PPI) have therapeutic potential in a range of disease states including neurodegenerative diseases (Parkinson's and Alzheimer's diseases), chronic obstructive pulmonary disease and various inflammatory conditions. By stalling Keap1‐mediated ubiquitination of Nrf2, such compounds can enhance Nrf2 transcriptional activity and activate the expression of a range of genes with antioxidant response elements in their promoter regions. Keap1 inhibitors based on peptide and small‐molecule templates have been identified. In this paper we develop the structure–activity relationships of the peptide series and identify a group of ligands incorporating unnatural amino acids that demonstrate improved binding affinity in fluorescence polarisation, differential scanning fluorimetry and isothermal titration calorimetry assays. These modified peptides have the potential for further development into peptidomimetic chemical probes to explore the role of Nrf2 in disease and as potential lead structures for drug development.

Increasing the activity of the transcription factor Nrf2 is an inducible cellular response to a range of inputs including redox and electrophilic stress and various intracellular stimuli.^1^ Nrf2 activation results in increased expression of a large battery of genes with antioxidant response elements (AREs) in their promoter regions.^2^ These include proteins associated with redox homeostasis (e.g., thioredoxin, thioredoxin reductase), phases I and II metabolism [e.g., NAD(P)H quinone oxidoreductase‐1 (NQO1), glutathione synthesis and conjugation enzymes] and proteins involved in autophagy (e.g., sequestosome‐1/p62, NDP52), amongst others.^1^, ^3^ Increasing Nrf2 activity has been proposed as a potential disease‐modifying intervention in neurodegenerative conditions such as Parkinson's and Alzheimer's disease and in various inflammatory conditions.^4^

The main negative regulators of Nrf2 activity are the proteins Keap1 and β‐TrCP, which target Nrf2 for ubiquitination and proteosomal degradation by interacting with degrons in the Neh2 and Neh6 domains of Nrf2, respectively.^5^ Keap1 is the major regulator and its interaction with Nrf2 has been studied extensively.^6^ Inhibition of the direct protein–protein interaction (PPI) between Keap1 and Nrf2 has been proposed as an intervention that can increase Nrf2 transcriptional activity by stalling its ubiquitination and turnover in the cell, thus prolonging its half‐life (Figure 1).^4^ Recently, several approaches to inhibition of the PPI between the C‐terminal Keap1 Kelch domain and the Neh2 domain of Nrf2 have been described.^7^ One of the early approaches to developing Keap1 inhibitors was based on the native peptide sequences of Nrf2—sequences **1** and **2**—and related proteins (p62, **3**, prothymosin‐α) that interact with the Kelch domain of Keap1.^8^ Such peptides have high affinities for Keap1, with the most active examples having IC_50_ values in the nanomolar range. Small‐molecule compounds based on sulfonamide scaffolds, such as compounds **4**, have been described, with **4 b** having demonstrated low‐nanomolar activity in competitive binding assays with Keap1.^9^ Other diverse structures identified through high‐throughput screening and SAR studies are generally less active.^10^ Most of these compounds are capable of inducing the expression of Nrf2 target genes in cells, although these effects are observed at micromolar concentrations in most cases.

**Figure 1:**
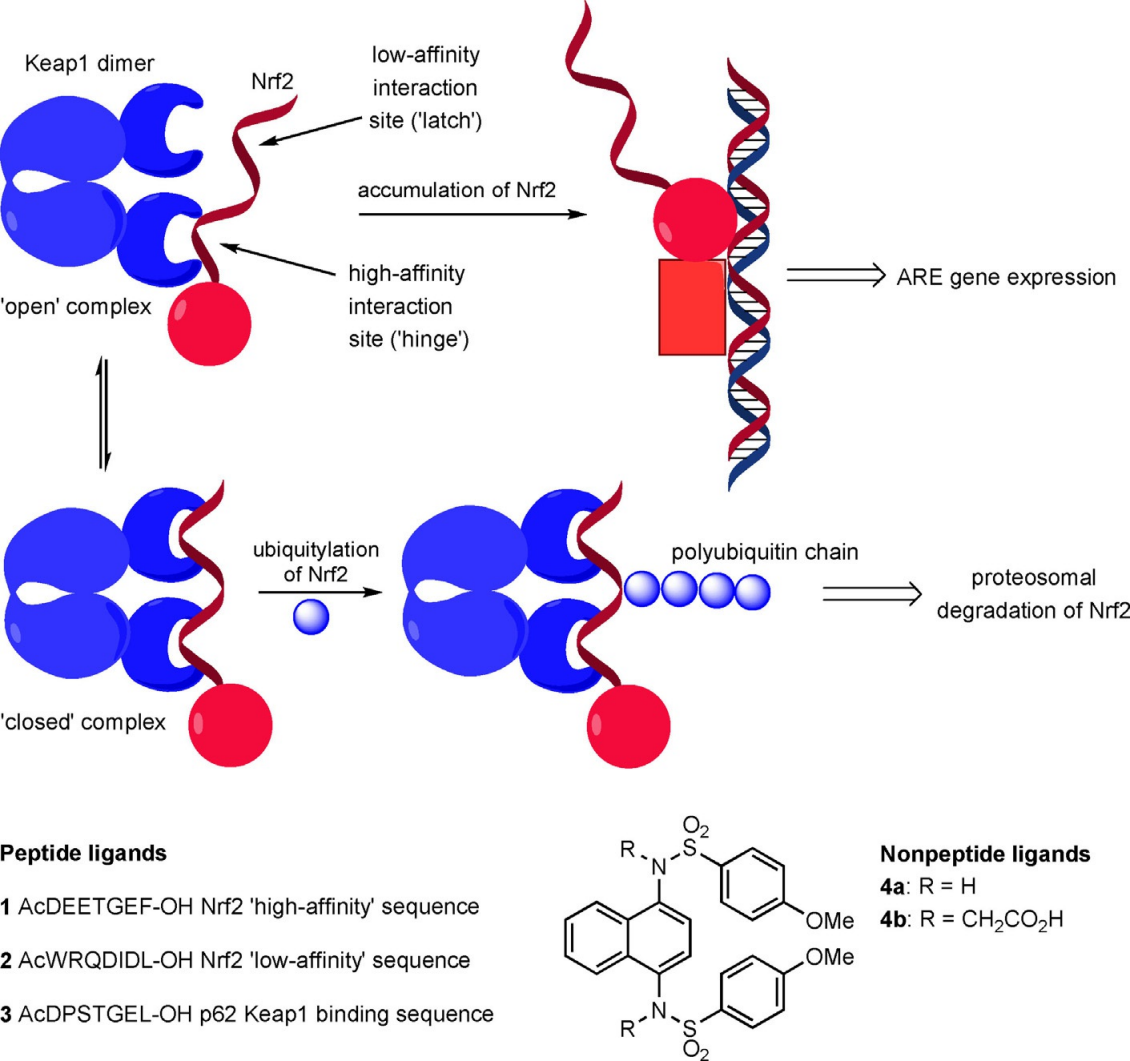
Schematic representation of the Keap1–Nrf2 “hinge and latch” interaction mechanism. Peptides **1** and **2** match the high‐ and low‐affinity sequences, respectively, of Nrf2, and **3** is derived from the Keap1 binding protein p62. Compounds **4** are examples of small‐molecule inhibitors of the Keap1–Nrf2 interaction.

Peptides represent orthogonal lead structures to known small‐molecule Keap1 inhibitors and have the potential to be developed into drug‐ or chemical‐probe‐like leads for further development. Indeed, co‐crystallisation studies have demonstrated that small‐molecule inhibitors and peptide ligands bind to subtly different conformations of the Keap1 protein, thus supporting the notion that divergent SAR profiles are feasible. We and others have found that peptides based upon the high‐affinity Nrf2 ETGE sequence have proved to be the most active in Keap1 binding assays, and hybrid sequences based upon the ETGE/p62 consensus sequences, such as **5**, have provided the best short (heptamer) interacting sequences.^8^, ^11^ However, these peptides are polar and have limited activity in cell‐based assays of Nrf2 induction. Modest enhancement of cellular activity can be achieved by conjugation of the peptides to fatty acids^12^ or TAT sequences;^13^ however, further development of the underlying peptide SAR is required in order to address the membrane permeability of the structures.

In this manuscript we describe some of our recent work in characterising the binding behaviour of short (heptameric) Keap1‐interactive peptides by X‐ray crystallography and in exploring the SAR of analogues that incorporate unnatural amino acids. We anticipate that any increases in binding could be used to offset removal or substitution of charged residues from the peptide sequence in subsequent compounds, thus progressing towards more cell‐permeable derivatives. We show that the incorporation of unnatural amino acids at key points in the sequence results in peptides with improved or maintained binding affinity [measured by fluorescence polarisation (FP), differential scanning fluorimetry (DSF) or isothermal titration calorimetry (ITC)] and we explain some of these changes by using in silico structural substitutions with use of the new crystal structures as templates.

We initially sought to investigate modifications on the C‐terminal α‐carboxylate group of the Nrf2/p62 hybrid peptide **5** (Table 1, below) with the aim of improving Keap1 binding activity and reducing the overall net charge of the series. Peptides bearing a C‐terminal *N*‐isopentyl amide or *N*‐benzyl amide moiety (peptides **6** and **7**, respectively) were prepared by nucleophilic cleavage of the common, fully deprotected, peptide precursor Ac‐DPETGEL from an HMBA resin. In addition, we synthesised the C‐terminal 1*H*‐tetrazole peptide **8** by using a standard Fmoc solid‐phase peptide synthesis (SPPS) procedure that included the direct attachment of Fmoc‐Leu‐T (**20**) to a 2‐chlorotrityl chloride resin as the loading step. The required building block **20** was prepared from the readily available Fmoc‐Leu‐OH (**17**) in a three‐step solution‐phase synthetic procedure analogous to that reported previously for the corresponding Cbz‐protected analogue.^15^ Briefly, **18** was converted into the primary amide analogue **19** by treatment with Boc_2_O and (NH_4_)_2_CO_3_ and then sequentially dehydrated with cyanuric chloride and subjected to [2+3] cycloaddition with NaN_3_ to furnish **21** in 55 % overall yield (Scheme 1).

**Scheme 1:**
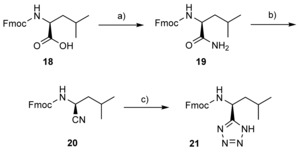
Synthetic route to 1*H*‐tetrazole **21**. a) (NH_4_)_2_CO_3_, Boc_2_O, pyridine, MeCN, 0 °C to RT, 16 h, 84 %. b) Cyanuric chloride, DMF, 0 °C to RT, 16 h, 98 %. c) NaN_3_, ZnBr_2_, H_2_O/*i*PrOH (2:1, *v*/*v*), reflux, 16 h, 67 %.

Previous work from our group has demonstrated that hydrophobic amino acids are generally well tolerated as C‐terminal residues in ETGE‐derived heptamer peptides.8a This prompted us to synthesise analogues of **5** in which leucine is replaced by non‐proteinogenic hydrophobic amino acids, because such modifications can increase the metabolic stability of peptides and lead to lipophilicity and potency improvements.^16^ Peptides incorporating *tert*‐leucine (Tle; peptide **9**), thienylalanine (Thi; peptide **10**) or cyclohexylalanine (Cha; peptide **11**) as C‐terminal residues were synthesised from commercially available *N*‐Fmoc‐protected amino acids by using standard SPPS procedures on a 2‐chlorotrityl chloride resin.

Additionally, we investigated modifications to the central threonine residue of the optimised peptide sequence. According to the available crystallographic and molecular docking data,^12^ the threonine side chain of the peptide occupies space above the central channel through the Keap1 Kelch domain with the methyl group pointing towards the pore. To explore the possibility of extending a portion of a modified side chain into this opening, we replaced threonine with the longer‐side‐chain amino acids homophenylalanine (hPhe; peptide **12**) and 2‐amino‐3‐benzamidopropanoic acid (Bap; peptide **13**). We also substituted threonine with asparagine (peptide **14**) to mimic the ENGE motif of the Keap1‐interacting protein prothymosin‐α.^17^

Peptides **12** and **14** were prepared from commercially available Fmoc‐protected amino acids, whereas **13** was synthesised by using a modified Fmoc‐SPPS procedure that included the on‐resin modification of the peptide precursor containing an *N*_β_‐trityl‐protected (*S*)‐2,3‐diaminopropanoic acid (Dap) residue at the appropriate position. The required building block **24** was prepared from Fmoc‐Asn‐OH (**22**) according to the synthetic sequence outlined in Scheme 2. Hoffmann rearrangement of **22** with [bis(trifluoroacetoxy)iodo]benzene afforded Fmoc‐Dap‐OH (**23**), which was in turn converted into **24** by installing temporary TMS protection on the α‐carboxylate group prior to treatment with trityl chloride. After the synthesis of the fully protected peptide precursor on resin, the DAP trityl group was selectively removed by treatment with a dilute solution of trifluoroacetic acid (TFA) in CH_2_Cl_2_/triisopropylsilane (TIS). The resulting free DAP amine was treated with benzoyl chloride to afford peptide **13** after simultaneous cleavage from the resin and global deprotection (Scheme 3).

**Scheme 2:**
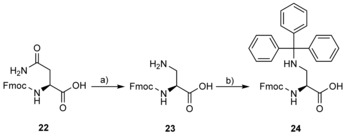
Synthetic route to the *N*_β_‐trityl‐protected Dap derivative **24**. a) [Bis(trifluoroacetoxy)iodo]benzene, pyridine, DMF/H_2_O (2:1, *v*/*v*), RT, 18 h, 86 %. b) TMSCl, CH_2_Cl_2_, reflux, 4 h, then TrtCl, pyridine, 0 °C to RT, 2 h, 77 %.

**Scheme 3:**
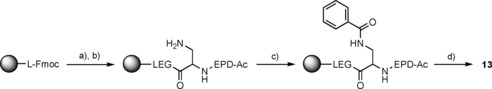
Synthetic route to peptide **13**. a) Fmoc‐SPPS cycles. b) TFA/TIS/CH_2_Cl_2_ (3:5:92, *v*/*v*/*v*), RT, 3×5 min. c) Benzoyl chloride, pyridine, DMF, RT, 5 h. d) TFA/TIS/H_2_O (95:2.5:2.5, *v*/*v*/*v*), RT, 3 h.

Additionally, we were interested in replacing proline with other cyclic amino acids in order to investigate how changes in the size and rigidity of the ring would affect the conformational stability of the peptide, as well as its binding affinity for the Keap1 Kelch binding pocket. Peptides incorporating the unnatural amino acids thiazolidine‐4‐carboxylic acid (thioproline, Thp; peptide **15**) or piperidine‐2‐carboxylic acid (Pip; peptide **16**) were prepared by following a standard Fmoc‐SPPS procedure with commercially available building blocks.

The ability of the peptides to interact with the Kelch domain of Keap1 was determined by use of a previously described FP assay.8a The native Nrf2 ETGE peptide **1** has an FP IC_50_ of 5.39 μm (calcd *K*_i_ 2.26 μm), whereas the Nrf2/p62 hybrid peptide **5** is more than 40 times more active. Replacing the C‐terminal carboxylate group of **5** either with an *N*‐isopentyl amide group (peptide **6**) or an *N*‐benzyl amide group (peptide **7**) led to a moderate drop in activity (Table 1). These results are consistent with a recently reported molecular modelling study in which the reduced activity of peptides containing C‐terminal amides was attributed to the absence of electrostatic interactions with Arg380.^18^ On the other hand, substituting the Cterminal carboxylate group with a 1*H*‐tetrazole moiety (peptide **8**), which has a similar p*K*_a_ value but is more lipophilic,^19^ was better tolerated than the previous modifications, although a threefold drop in activity in relation to the parent peptide **5** was recorded.

**Table 1:** FP IC_50_ and DSF Δ*T*_m_ values for Keap1 peptide inhibitors.

Cpd^[a]^	Sequence	FP IC_50_ [nm]	FP calcd *K*_i_ [nm]	Δ*T*_m_ [°C]
**1**	Ac‐Asp‐Glu‐Glu‐Thr‐Gly‐Glu‐Phe‐OH^[a]^	5390±580^[a]^	2265	1.95±0.1
**5**	Ac‐Asp‐Pro‐Glu‐Thr‐Gly‐Glu‐Leu‐OH^[a]^	115±13^[a]^	48	n.d.^[b]^
**6**	Ac‐Asp‐Pro‐Glu‐Thr‐Gly‐Glu‐Leu‐NH‐isopentyl	745±126	313	2.0±0.1
**7**	Ac‐Asp‐Pro‐Glu‐Thr‐Gly‐Glu‐Leu‐NH‐Bn	888±28	373	2.5±0.1
**8**	Ac‐Asp‐Pro‐Glu‐Thr‐Gly‐Glu‐Leu‐TET	374±31	157	6.2±0.3
**9**	Ac‐Asp‐Pro‐Glu‐Thr‐Gly‐Glu‐Tle‐OH	235±16	99	5.8±0.6
**10**	Ac‐Asp‐Pro‐Glu‐Thr‐Gly‐Glu‐Thi‐OH	578±25	243	5.3±0.2
**11**	Ac‐Asp‐Pro‐Glu‐Thr‐Gly‐Glu‐Cha‐OH	85±14	36	5.8±0.9
**12**	Ac‐Asp‐Pro‐Glu‐hPhe‐Gly‐Glu‐Leu‐OH	20 %^[c]^	n.d.^[b]^	0.8±0.2
**13**	Ac‐Asp‐Pro‐Glu‐Bap‐Gly‐Glu‐Leu‐OH	27 %^[c]^	n.d.^[b]^	0.5±0.1
**14**	Ac‐Asp‐Pro‐Glu‐Asn‐Gly‐Glu‐Leu‐OH	3036±310	1276	0.7±0.2
**15**	Ac‐Asp‐Thp‐Glu‐Thr‐Gly‐Glu‐Leu‐OH	89±6	37	8.3±0.3
**16**	Ac‐Asp‐Pip‐Glu‐Thr‐Gly‐Glu‐Leu‐OH	1063±280	447	1.8±0.2
**17**	Ac‐Asp‐Thp‐Glu‐Thr‐Gly‐Glu‐Cha‐OH	31±3.7	13	n.d.^[b]^

[a] Data from Hancock et al.8a [b] Not determined. [c] Percentage inhibition at 100 μm concentration of inhibitor. Estimated *K*_i_ values were calculated by the method described by Kenakin.^14^

In addition to the changes in the α‐carboxylate group of leucine, we had also synthesised a small set of peptides bearing modified side chains at the C terminus. Replacing leucine with *tert*‐leucine (peptide **9**), with a shorter but bulkier side chain, resulted in twofold lower activity, whereas the aromatic thienylalanine (peptide **10**) was less well tolerated at this position, giving a fivefold drop in binding affinity. Interestingly, changing the isobutyl side chain group of leucine to a cyclohexylmethyl system (peptide **11**) led to a moderate potency improvement (FP IC_50_ 85 nm, calcd *K*_i_ 36 nm), an effect that could be attributed to an increase in hydrophobic contacts with the Keap1 binding pocket.

Replacement of threonine with the longer‐side‐chain amino acids homophenylalanine (peptide **12**) and 2‐amino‐3‐benzamidopropanoic acid (peptide **13**) resulted in a dramatic drop in activity that mirrors the previously reported effects of Ala or Val substitution.^11^ On the other hand, substitution of threonine with asparagine (peptide **14**), which is present in the ENGE motif of the Keap1‐interacting protein prothymosin‐α,^17^ was better tolerated; however, the binding affinity of the corresponding peptide **14** was reduced by more than one order of magnitude in relation to that of **5**. These results are consistent with a recent molecular modelling study suggesting that despite its similarity to the Nrf2 ETGE motif, the Keap1‐interacting region of prothymosin‐α is significantly more disordered.^20^

Substituting l‐thiazolidine‐4‐carboxylic acid for proline (peptide **15**) resulted in an increased Keap1 binding activity and an FP IC_50_ of 89 nm (calcd *K*_i_ 37 nm). The Thp thiazolidine ring has a rigidity and size different from those of the pyrrolidine ring of proline,^21^ and its incorporation into short peptides has been shown to induce a stabilisation of the *cis*‐amide conformation, effects that could account for the recorded affinity improvement.^22^ In contrast, changing proline to (*S*)‐piperidine‐2‐carboxylic acid (peptide **16**) resulted in a more than tenfold drop in binding activity (Table 1). Incorporating two unnatural amino acids in the peptide sequence (peptide **17**) resulted in an improvement in the binding to Keap1 (IC_50_ 31 nm, calcd *K*_i_ 13 nm), thus suggesting that the substitutions might be partially additive in their effects on binding activity.

The binding activity of peptides **6**–**16** against the Keap1 Kelch domain was further characterised in a secondary DSF screening assay at a fixed concentration of 10 μm (Table 1, Figure 2). The midpoint temperature of transition (*T*_m_) for the thermal denaturation of the Keap1 Kelch protein was estimated to be (47.0±0.1) °C, whereas the Nrf2 ETGE peptide **1**, used as a positive control, induced a shift in the *T*_m_ (Δ*T*_m_) of (1.5±0.2) °C. Consistent with their weak binding profile in the FP assay, peptides **12**, **13** and **14** caused only a marginal thermal stabilisation (Δ*T*_m_ of ≈0.5 °C) of the Keap1 Kelch protein in the DSF screen, whereas **6**, **7** and **16**, which have FP IC_50_ values in the sub‐micromolar to low‐micromolar concentration range, were considerably more active (Δ*T*_m_ of ≈2 °C). On the other hand, peptides **8**–**11** had a more profound effect on the Keap1 Kelch *T*_m_ (Δ*T*_m_ of ≈5–6 °C), which is in agreement with their increased potency as recorded in the FP assay. Interestingly, peptide **15** induced a Δ*T*_m_ of 8.3 °C, thus confirming its improved binding affinity in relation to the other analogues of this series. Although it is difficult to compare the DSF and FP binding data directly (fixed concentration and variable concentration studies, respectively), there is a reasonable correlation between the two sets of data (*R* ^2^=0.743 with use of a logarithmic fit; Figure S1 in the Supporting Information).

**Figure 2:**
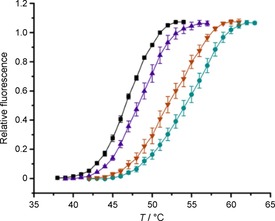
DSF melt curves for the Keap1 Kelch protein in the presence of DMSO (control, ▪) or 10 μm concentrations of **1** (▴), **8** (▾) or **15** (•), *n*=4.

Motivated by the promising binding profiles observed in the FP and DSF assays, we sought additional confirmation of the binding activity of the peptides through ITC. In agreement with our previous report,8a peptide **5** had an ITC *K*_d_ of 250 nm (Figure 3 A, Table 2), consistent with its FP IC_50_ of 115 nm (Table 1). Analysis of the thermodynamic profile of the interaction demonstrated a binding event that is enthalpy‐driven (Δ*H*), with a small entropic (*TΔS*) penalty, possibly reflecting the stabilisation of the β‐hairpin conformation of the peptide by the proline residue. On the other hand, replacing leucine of peptide **5** with *tert*‐leucine in **9** led to an approximately twofold drop in binding affinity that was characterised by an increased entropic penalty (Figure 3 B, Table 2). In agreement with the previously described FP data, peptide **15** showed a good binding profile in the ITC assay with a calculated *K*_d_ of 310 nm (Figure 3 C, Table 2). Peptide **11**, with an FP IC_50_ of 89 nm, gave a comparable ITC *K*_d_ of 75 nm whereas its analogue **17**, incorporating both a thioproline and cyclohexylalanine residue, had a *K*_d_ of 56 nm, similar to its FP IC_50_ of 31 nm. In relation to peptide **5**, improvements in the binding enthalpy recorded for **15** were offset by negative changes in the entropy of binding, resulting in a similar binding free energy (Δ*G*). The apparent large enthalpy/entropy compensation for peptide **17** warrants further investigation.

**Figure 3:**
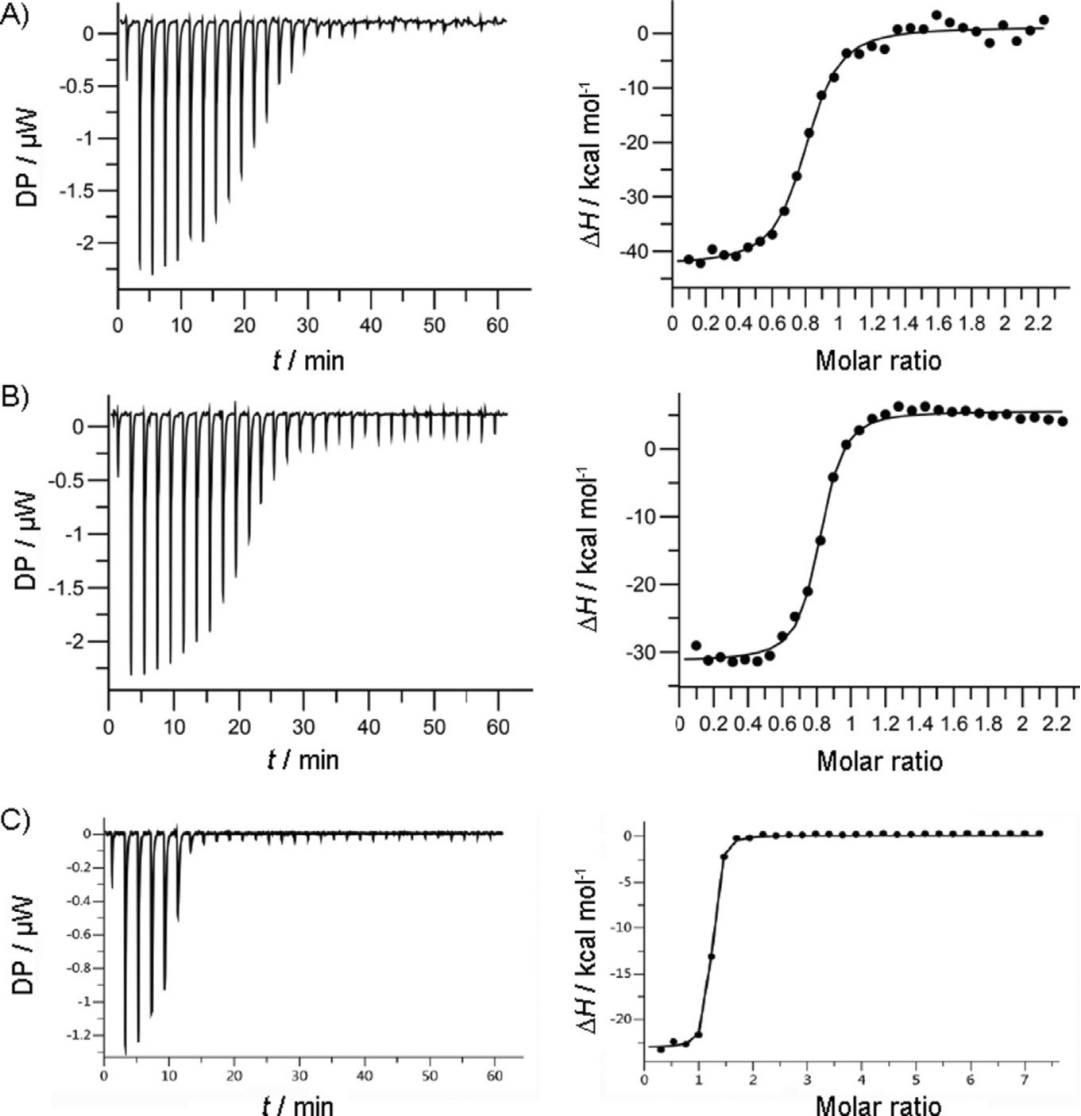
ITC analyses of peptide binding to Keap1. Raw (left) and normalised (right) ITC data for titrations plotted versus the inhibitor/protein molar ratio demonstrating saturable exothermic reaction upon sequential additions of A) **5**, B) **9**, and C) **17** at 500 μm.

**Table 2:** Thermodynamic parameters extracted from the calorimetric evaluation of Keap1 binding with different peptides (*n*≥2, *T*=25 °C).

Cpd	ITC *K*_d_ [μm]	Δ*G* [kcal mol^−1^]	Δ*H* [kcal mol^−1^]	*T*Δ*S* [kcal mol^−1^]
**5**	0.25±0.10	−9.03±0.07	−9.07±0.35	−0.03±0.28
**9**	0.58±0.09	−8.59±0.12	−10.25±0.21	−1.69±0.34
**11**	0.075±0.006	−9.57±0.21	−9.83±0.22	−0.29±0.001
**15**	0.31±0.01	−8.95±0.17	−12.83±0.33	−3.84±0.31
**17**	0.056±0.005	−9.89±0.04	−23.2±0.21	−13.25±0.07

To date, co‐crystallisation studies with Keap1 and peptides have used relatively long linear sequences of 14–16 amino acids,^17^, ^23^ as well as one 34‐mer sequence,^24^ with only one example of a shorter cyclic heptameric^25^ Nrf2‐derived peptide. We soaked human Keap1 Kelch domain crystals with peptides **1** and **5** to determine their bound conformations, with the aim of observing the effect of shortening the N and C termini on the conformation of the peptide. The resulting crystal structures had resolutions of 2.92 Å (peptide **1**, PDB ID: 6FMP) and 2.10 Å (peptide **5**, PDB ID: 6FMQ). In each case the peptide occupied the binding pocket of one of the two Keap1 proteins in the unit cell (Figure S1 a, b) and entered into interactions with the vacant Keap1 Kelch domain through polar interactions between the C‐terminal carboxylate group of the peptide and Arg380 of the adjacent protein (Figure S1 c, d). Otherwise, the peptide are oriented in a similar manner to the previously described ETGE 16‐mer peptide23a (Figure 4 A, B), with the exception of the Asp side chain being positioned differently due to rotation about the Cα−CO bond.

**Figure 4:**
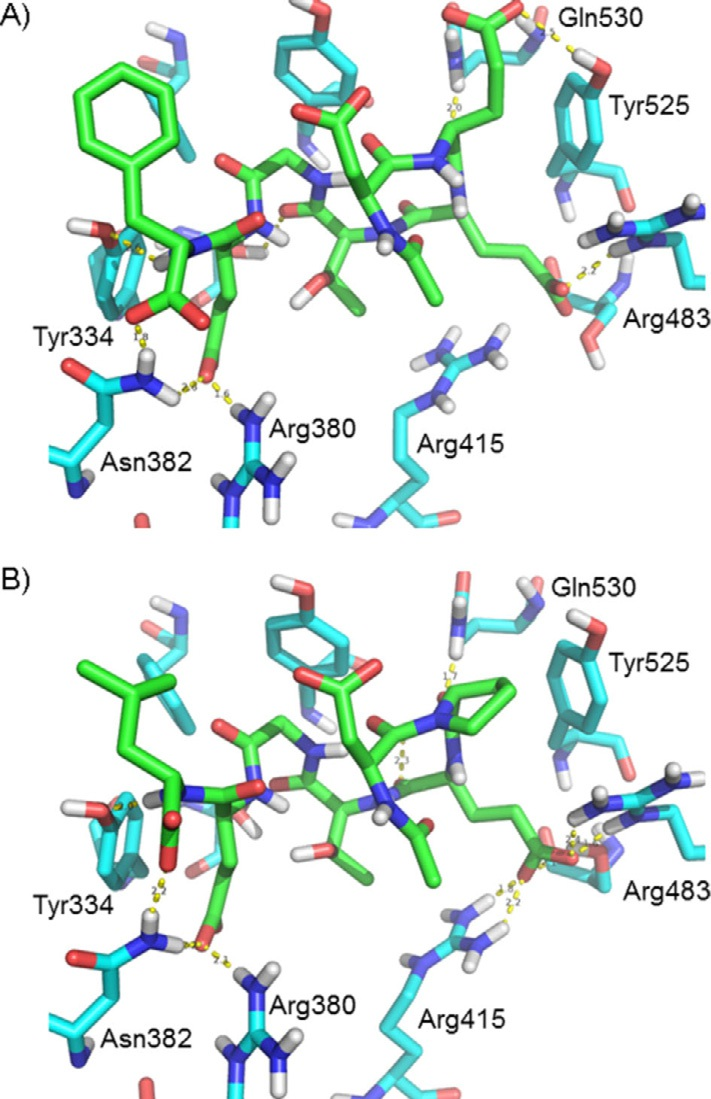
Interactions between peptides **1** and **5** and the Keap1 Kelch domain. A) Peptide **1⋅**Keap1 Kelch domain (PDB ID: 6FMP) and B) peptide **5⋅**Keap1 Kelch domain (PDB ID: 6FMQ) structures; selected protein residues are shown in cyan and peptide residues in green.

Subsequently, we examined the crystal structures to determine how the various structural changes made in our current study might be accommodated in the binding pocket (Figure 4). Peptide **1** is involved in electrostatic and/or hydrogen‐bond interactions with Arg380, Asn382, Arg483, Gln530, Tyr525, and Ser602 (Figure 4 A), whereas peptide **5** has an additional interaction with Arg415, but lacks the glutamate side chain that interacts with Tyr525 (Figure 4 B). Peptide **5** shows a tighter interaction with Keap1, probably due to the conformational restriction introduced by the Glu>Pro substitution, which restricts the mobility of the peptide backbone. In silico structural replacements within the crystal structure to convert **5** into **8** (C‐terminal carboxylate group to tetrazole) suggest that the larger tetrazole, in its deprotonated form, can occupy a similar space and could engage in hydrogen‐bond interactions with Asn382 (Figure 5 A; cf. 5 B). This is consistent with its comparable binding affinity to Keap1 observed in the FP and ITC experiments (Tables 1 and 2). Similarly, the larger thioproline residue present in **15** could be accommodated in place of proline without clashing with the edge of the binding pocket (Figure 5 C; cf. 5 D). The limited structural distortion resulting from the changing of the proline to thioproline suggests that the network of polar interactions formed by **15** in the modified structure could be similar to those formed by the other peptides, so the nature of the different enthalpy and entropy contributions from **15** versus **5** in the ITC study (Table 2) requires further investigation.

**Figure 5:**
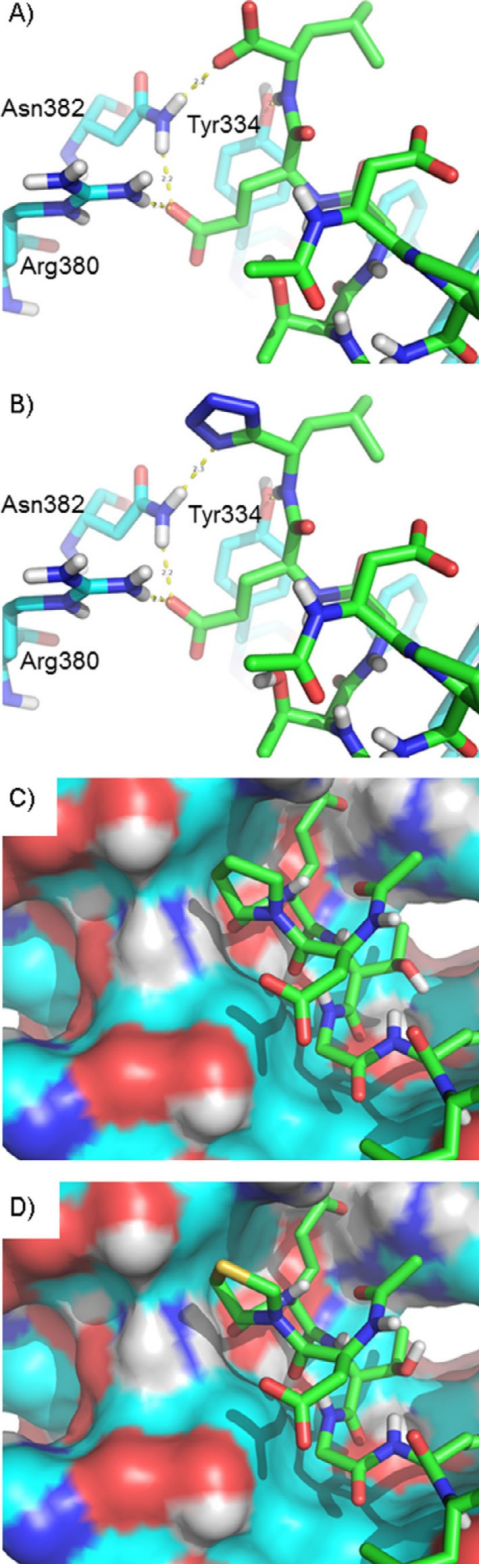
Bound conformations of peptides in complex with the Keap1 Kelch domain. A) Peptide **5** participates in polar interactions through its C‐terminal carboxylate group. B) The modelled conformation of peptide **8** suggests that a tetrazole unit can mimic such interactions. C) The proline residue of peptide **5** occupies space near the surface of the binding pocket. D) The modelled conformation of peptide **15** suggests that the thioproline reside can be accommodated at this site. Peptides are represented as green sticks, Keap1 is represented as cyan sticks with selected residues A), B) labelled, or C), D) as a surface, and polar interactions are shown as yellow dotted lines.

The new peptide structures described in this study provide further insights into the structural requirements for binding of molecules of this class to Keap1. It is notable that the structural changes that improve or maintain binding affinity are relatively modular: proline to thioproline (**5** vs. **15**), leucine to *tert*‐leucine or cyclohexylalanine (**5** vs. **8** or **11**) and C‐terminal carboxylate group to tetrazole (**5** vs. **8**). Thus, combining two or more of these changes in a single compound might be advantageous. The improved activity of peptide **17**, which incorporates both a thioproline and a cyclohexylalanine residue, appears to support this. We would anticipate that incorporating extensions of the N‐terminal acetyl group (e.g., to steroyl) as we described previously^12^ would be expected to yield a peptide with improved binding affinity and, potentially, improved biological stability and permeability.

## Experimental Section

All synthetic procedures, characterisation data for all new compounds (^1^H and ^13^C NMR, HPLC, LC‐MS, HRMS) and biophysical techniques (FP and DSF assays, ITC, and molecular modelling) are detailed in the Supporting Information.

## Conflict of interest

*G.W., J.G., and N.D.G. are founders of Keregen Therapeutics, Ltd, an SME with an interest in developing small‐molecule inducers of Nrf2*.

## Supporting information

As a service to our authors and readers, this journal provides supporting information supplied by the authors. Such materials are peer reviewed and may be re‐organized for online delivery, but are not copy‐edited or typeset. Technical support issues arising from supporting information (other than missing files) should be addressed to the authors.

Supplementary
