# Supplementary material for: Modified Peptide Inhibitors of the Keap1–Nrf2 Protein–Protein Interaction Incorporating Unnatural Amino Acids
Source: Chembiochem. 2018 Jul 18;19(17):1810–6. doi: 10.1002/cbic.201800170 (PMC6220877; doi:10.1002/cbic.201800170)
Supplement: Supplementary file 1 — Supplementary [file CBIC-19-1810-s001.pdf]

CHEMBIOCHEM

Supporting Information

Modified Peptide Inhibitors of the Keap1–Nrf2 Protein–Protein Interaction Incorporating Unnatural Amino Acids

Nikolaos D. Georgakopoulos, Sandeep K. Talapatra, Jemma Gatliff, Frank Kozielski, and Geoff Wells*[a]

cbic_201800170_sm_miscellaneous_information.pdf

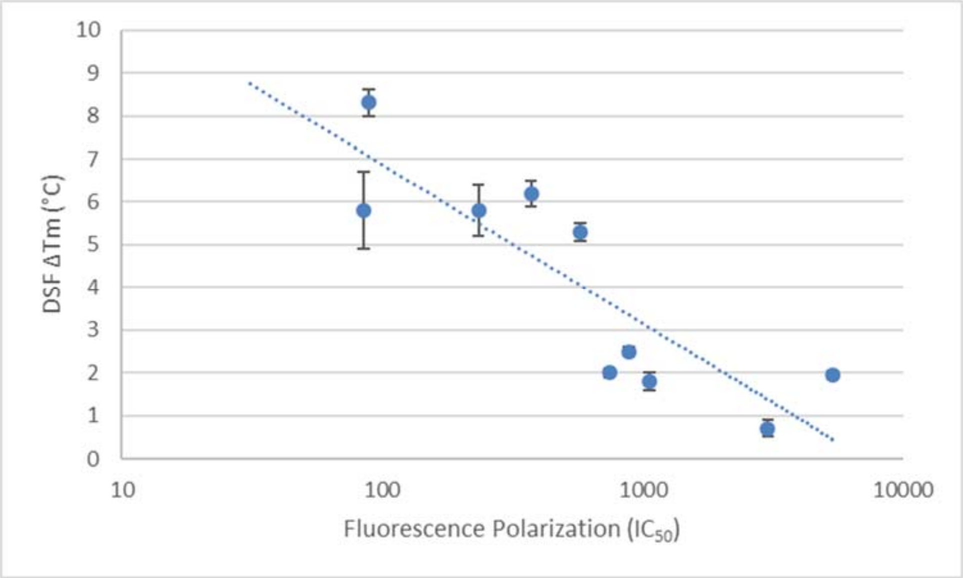

Figure S1. Correlation between the peptide fluorescence polarization IC_{50} values and the DSF ΔT_m values for binding to the Keap1 Kelch domain presented in Table 1. The logarithmic fit has the equation $y = -1.61\ln(x) + 14.285$ and the coefficient of determination $R^2 = 0.743$.

Experimental Section

Synthetic methods

All anhydrous solvents and reagents were purchased from commercial suppliers (e.g. Sigma Aldrich UK, Alfa Aesar, Acros, VWR or Fisher UK) and were used without further purification. All α-amino acids had the L-configuration (unless stated) and fluorenylmethoxycarbonyl (Fmoc) was used for *N*α protection. Peptides **11** and **17** were purchased from Peptide Protein Research Ltd, Fareham, UK.

^{1}H NMR spectra were recorded at ambient temperature in [D$_6$]DMSO on a Bruker Advance 400 Spectrophotometer at 400.13 MHz or Bruker Advance 500 Spectrophotometer (cryoprobe) at 500 MHz. Chemical shifts are reported in parts per million (ppm) downfield from the tetramethylsilane reference (δ = 0) using the residual protonated solvent as an internal standard (^{1}H: δ ([D$_6$]DMSO) = 2.50 ppm). Data for ^{1}H NMR is given as follows: chemical shift (multiplicity, coupling constants (*J*, given in Hertz (Hz)), integration and assignment). Multiplicities in the ^{1}H NMR spectra are quoted as: s = singlet, d = doublet, t = triplet, q = quartet, m = multiplet, dd = double doublet, ddd = double double doublet or td (triplet of

doublets). Splitting patterns that could not be interpreted or easily visualised were recorded as multiplets (m) or broad peaks (br). ^{13}C NMR spectra were recorded at ambient temperature in [D_6]DMSO on a Bruker Advance 400 Spectrophotometer at 100.61 MHz, or Bruker Advance 500 Spectrophotometer (cryoprobe) at 125 MHz. Chemical shifts were measured in ppm relative to tetramethylsilane (δ = 0) and referenced to the [D_6]DMSO residual dimethyl sulphoxide signal δ ([D_6]DMSO) = 39.4 ppm. High-resolution mass spectrometry (HRMS) spectra were obtained from UCL School of Pharmacy, Structural Chemistry, on a Micromass Q-TOF Premier Tandem Mass Spectrometer coupled to an HPLC instrument using electrospray ionisation (ESI) mass spectrometry. *m/z* values are reported in Daltons. LC-MS spectra were recorded using a Shimadzu LCMS-2020 equipped with an XTerra® MS C18 column (4.6 x 50 mm, 2.5 µm) and a flow rate of 0.6 mL/min. The eluent system consisted of eluent A (H_2O with 0.1% formic acid, HPLC grade) and eluent B (MeCN with 0.1% formic acid, HPLC grade) with the following conditions: 0.0 – 2.5 min 90% A: 10% B, then a linear gradient from 2.5 min to 5.5 min to a final composition of 5% A: 95% B that was maintained for a further 2.5 min, then adjusted to 10% A: 90% B over 10.5 min and held for 1.5 min. Total run time = 12 min.

SPPS general procedures

Peptides were synthesised using standard manual Fmoc solid phase chemistry. In each case, the appropriate resin was selected according to the required *C*-terminal functionality. Rink amide resin was used for the preparation of *C*-terminal carboxamide peptides, while 2-chlorotrityl chloride or Wang resin was employed for *C*-terminal carboxylates. 2-Chlorotrityl chloride resin was also used for the synthesis of peptides with a *C*-terminal 1*H*-tetrazol-5-yl functionality. Hydroxymethylbenzoic acid (HMBA) resin was selected for the preparation of peptides with a C-terminal secondary amide group. Attachment of the first amino acid to the solid support was the initial synthetic step when using 2-chlorotrityl chloride or HMBA resin, while in the case of Rink amide or Wang resin, removal of the Fmoc protecting group was required before loading the first amino acid. Peptide elongation was then performed through sequential amino acid couplings and Fmoc deprotection steps. The N-terminus was capped with an acetyl group by treatment with Ac_2O. Following a simultaneous side-chain deprotection and resin-cleavage, the peptides were subsequently isolated and purified.

Coupling of the first amino acid to rink resin: The resin was placed in a solid phase synthesis tube, washed with DCM (3 x 5 mL) and DMF (3 x 5 mL) and drained. DMF (5 mL) was added and the resin was allowed to swell at RT for 30 min. The Fmoc protecting group was removed using 20% piperidine in DMF and the first amino acid was then coupled using HATU (2.5 eq. relative to resin loading) and DIPEA (5 eq. relative to resin loading) in DMF. The resin was

washed thoroughly with DMF (3 x 5 mL), DCM (3 x 5 mL) and again DMF (5 mL) before continuing the synthesis.

Coupling of the first amino acid to 2-chlorotrityl chloride resin: The resin was allowed to swell in dry DCM (5 mL) at RT for 30 min. The first amino acid (1.5 eq. relative to resin loading) and DIPEA (5 eq. relative to the amino acid) were dissolved in dry DCM (5 mL) and the solution was added to the resin. The mixture was agitated for 5 min. Following the addition of further 1.5 eq. of DIPEA (relative to the amino acid), the mixture was agitated vigorously for 60 min. The resin was washed with DCM (3 x 5 mL) and MeOH (0.8 mL per gram of resin) was used to endcap any remaining reactive 2-chlorotrityl groups. The resin was washed thoroughly with DMF (3 x 5 mL), DCM (3 x 5 mL) and again DMF (5 mL) before continuing the synthesis.

Coupling of the first amino acid to HMBA resin: Fmoc-Leu-OH (**14**) (10 eq. relative to resin loading) was dissolved in DCM (4.5 mL). Two drops of DMF were added to aid dissolution and the reaction was cooled to 0-5°C. *N,N'*-Diisopropylcarbodiimide (5 eq. relative to resin loading) was dissolved in dry DCM and added to the reaction mixture, which was then stirred for 20 min at 0-5°C under a $CaCl_2$ drying tube. The solvent was removed by evaporation and the resulting anhydride was used without further purification in the next step. In an SPPS reaction tube, the resin was allowed to swell in dry DMF (5 mL) at RT for 30 min. The resin was subsequently washed with DMF (3 x 5 mL) and DCM (3 x 5 mL). The Fmoc-Leu-anhydride was added to the resin along with DMAP (0.1 eq.) in DMF (5 mL). The mixture was agitated for 60 min and the procedure was repeated once. The resin was washed thoroughly with DMF (3 x 5 mL), DCM (3 x 5 mL) and again with DMF (5 mL) before continuing the synthesis.

Removal of Fmoc-protecting group: A solution of 20% piperidine in DMF (5 mL) was added to the resin and the reaction was agitated at RT for 7 min. The resin was drained and washed with DMF (3 x 5 mL) and DCM (3 x 5 mL). This procedure was repeated three times

General amide coupling method: The Fmoc amino acid (2.5 eq. relative to the resin loading), HATU (2.5 eq. relative to resin loading) and DIPEA (5 eq. relative to resin loading) were dissolved in DMF (5 mL). This solution was added to the resin and agitated for 1 h. The resin was drained and washed with DMF (3 x 5 mL) and DCM (3 x 5 mL). The procedure was repeated once.

N-terminal acetylation: Acetic anhydride (5 eq. relative to resin loading), DIPEA (10 eq. relative to resin loading) and DMF (3 mL) were added to the resin and mixed for 1 h at RT. The resin was drained and washed with DMF (3 x 5 mL) and DCM (3 x 5 mL). The procedure was repeated once.

Removal from the resin and side chain deprotection: The resin was washed with DMF (3 x 5 mL), DCM (3 x 5 mL) and methanol (3 x 5 mL) and dried overnight in vacuo. The resin was transferred to a conical flask and 20 mL of cleavage cocktail was added (standard cleavage cocktail: TFA/TIS/H_2O 95:2.5:2.5 *v/v/v* or Reagent K: TFA/phenol/H_2O/thioanisole/EDT 82.5:5:5:5:2.5 *v/v/v/v/v*). The mixture was allowed to stand at RT for 2-3 h. The resin was filtered, washed with a small amount of TFA and the combined filtrates were concentrated in vacuo.

Peptide isolation and purification: Ice-cold Et_2O (30-35 mL) was added to the concentrated sample to aid precipitation of the crude product. The suspension was centrifuged at 2000 *g* for 5 min and the ether was decanted. This procedure was repeated twice. The resulting pellet was dried overnight in vacuo.

HPLC methods

Analytical reversed-phase HPLC was carried out on an XSELECT™ CSH™ C_{18} column 50 x 6 mm (particle size: 2.5 μm) at a flow rate of 1.0 mL/min. The eluent system consisted of eluent A (H_2O with 0.1% TFA, HPLC grade) and eluent B (MeCN with 0.1% TFA, HPLC grade) with the following gradient conditions: System A – Initial fixed composition 5% B to 50% B over 20 min, then increased to 95% B over 2 min, held for 2 min at 95% B, then returned to 5% B in 1 min. Total duration of gradient run was 25 min.

Preparative reversed-phase HPLC was carried out on an XSELECT™ CSM™ C_{18} column 250 x 10 mm (particle size: 2.5 μm) at a flow rate of 5.0 mL/min. The eluent system consisted of eluent A (H_2O with 0.1% TFA, HPLC grade) and eluent B (MeCN with 0.1% TFA, HPLC grade) with the following gradient conditions:

System B – Initial fixed composition 5% B to 15% B over 60 min, then 15% B to 95% B over 1 min, held at 95% B for 3 min, returned to 5% B in 1 min and held for 1 min. Total duration of gradient run was 66 min.

System C – Initial fixed composition 5% B to 14% B over 45 min, then 14% B to 95% B over 1 min, held at 95% B for 3 min, returned to 5% B in 1 min and held for 1 min. Total duration of gradient run was 51 min.

System D – Initial fixed composition 5% B to 16% B over 45 min, then 16% B to 95% B over 1 min, held at 95% B for 3 min, returned to 5% B in 1 min and held for 1 min. Total duration of gradient run was 51 min.

System E – Initial fixed composition 5% B to 16% B over 48 min, then 16% B to 95% B over 1 min, held at 95% B for 3 min, returned to 5% B in 1 min and held for 1 min. Total duration of gradient run was 54 min.

System F – Initial fixed composition 5% B to 27% B over 45 min, then 27% B to 95% B over 1 min, held at 95% B for 3 min, returned to 5% B in 1 min and held for 1 min. Total duration of gradient run was 51 min.

System G – Initial fixed composition 5% B to 30% B over 46 min, then 30% B to 95% B over 1 min, held at 95% B for 3 min, returned to 5% B in 1 min and held for 1 min. Total duration of gradient run was 45 min.

System H – Initial fixed composition 5% B to 25% B over 40 min, then 25% B to 95% B over 1 min, held at 95% B for 3 min, returned to 5% B in 1 min and held for 1 min. Total duration of gradient run was 46 min.

System I – Initial fixed composition 5% B to 18% B over 40 min, then 18% B to 95% B over 1 min, held at 95% B for 3 min, returned to 5% B in 1 min and held for 1 min. Total duration of gradient run was 46 min.

System J – Initial fixed composition 5% B to 21% B over 41 min, then 21% B to 95% B over 1 min, held at 95% B for 3 min, returned to 5% B in 1 min and held for 1 min. Total duration of gradient run was 47 min.

Compound 6: Ac-Asp-Pro-Glu-Thr-Gly-Glu-Leu-NH-*i*Pentyl

HMBA resin (100 mg, 0.93 mmol/g) was used to prepare the resin bound precursor Ac-Asp(OtBu)-Pro-Glu(OtBu)-Thr(tBu)-Gly-Glu(OtBu)-Leu-Wang resin according to the general procedure. The acid-sensitive side chain protecting groups of Asp, Glu and Thr were removed using a deprotection cocktail that consisted of TFA/TIS/H_2O (95:2.5:2.5 *v/v/v*). The resin was swelled with THF (4 mL) for 1 h. A cleavage solution of TEA/isopentylamine/THF (5 mL, 1:5:5 *v/v/v*) was added and the reaction was stirred overnight at 50°C. The solution was filtered and the resin was washed first with isopentylamine/THF and then with DCM (3 x 5 mL each). The filtrate was concentrated to a volume of ~ 1 mL and the crude material precipitated by the addition of ice-cold Et_2O. The peptide was isolated, subjected to preparative HPLC separation (system B) and lyophilised to give a white powder.

HRMS (ESI): calculated for $C_{38}H_{62}N_8O_{15}$ $[M{-}H]^-$ 869.4257, found 869.4219. LC-MS: *m/z* (ESI)= 869.20 $[M{-}H]^-$, t_R= 4.50 min, purity: >95%. HPLC retention time (system B): t_R= 48.39

min; (system A): t_R= 11.26 min, purity: >95%. Mass of crude product: 50 mg. Pure peptide obtained from 50 mg of crude: 3 mg.

Compound 7: Ac-Asp-Pro-Glu-Thr-Gly-Glu-Leu-NH-Bn

HMBA resin (300 mg, 0.93 mmol/g) was used to prepare the resin bound precursor Ac-Asp(OtBu)-Pro-Glu(OtBu)-Thr(tBu)-Gly-Glu(OtBu)-Leu-Wang resin according to the general procedure. The acid-sensitive side chain protecting groups of Asp, Glu and Thr were removed using a deprotection cocktail that consisted of TFA/TIS/H_2O (95:2.5:2.5 *v/v/v*). The resin was swelled with THF (4 mL) for 1 h. A cleavage solution of TEA/benzylamine/THF (15 mL, 1:5:5 *v/v/v*) was added and the reaction was stirred overnight at 50°C. The solution was filtered and the resin was washed first with benzylamine/THF and then with DCM (3 x 5 mL each). The filtrate was concentrated to a volume of ~ 1 mL and the crude material precipitated by the addition of ice-cold Et_2O. The peptide was isolated, subjected to preparative HPLC separation (system B) and lyophilised to give a white powder.

^{1}H NMR (500 MHz, [D$_6$]DMSO) δ (ppm): 12.59 – 11.81 (br, 3H), 8.38 (dd, J = 6.0, 5.9 Hz, 1H), 8.28 (d, J = 7.9 Hz, 1H), 8.06 – 7.93 (m, 4H), 7.35 – 7.19 (m, 5H), 7.51 (d, J = 7.9 Hz, 1H), 4.81 (dd, J = 13.7, 8.1 Hz, 1H), 4.38 – 4.09 (m, 7H), 4.04 – 3.90 (m, 1H), 3.83 – 3.35 (m, 5H), 2.67 (dd, J = 16.6, 5.5 Hz, 1H), 2.42 (dd, J = 16.6, 8.1 Hz, 1H), 2.38 – 2.17 (m, 4H), 2.12 – 1.38 (m, 14H), 1.03 (d, J = 6.3 Hz, 3H), 0.85 (dd, J = 27.4, 6.5 Hz, 6H). ^{13}C NMR (125 MHz, [D$_6$]DMSO) δ (ppm): 174.1, 174.0, 171.8, 171.6, 171.2, 170.8, 170.3, 169.5, 168.9, 168.8, 139.3, 128.2, 127.0, 126.7, 66.6, 59.5, 58.2, 52.1, 51.8, 51.2, 47.4, 46.8, 42.1, 41.9, 40.7, 36.0, 30.2, 30.1, 29.1, 27.5, 26.5, 24.3, 24.2, 22.9, 22.2, 21.6, 19.4. The product exists as a (major/minor 3:1) mixture of rotamers. HRMS (ESI): calculated for $C_{40}H_{58}N_8O_{15}$ $[M+H]^+$ 891.4100, found 891.4095. LC-MS: *m/z* (ESI)= 889.90 $[M-H]^-$, t_R= 4.31 min, purity: >95%. HPLC retention time (system B): t_R= 54.31 min; (system A): t_R= 10.69 min, purity: >95%. Mass of crude product: 189 mg. Pure peptide obtained from 100 mg of crude: 19 mg.

Compound 8: Ac-Asp-Pro-Glu-Thr-Gly-Glu-Leu-TET

2-Chlorotrityl chloride resin (500 mg, 1.70 mmol/g) was used to prepare this compound according to the general methods. The crude material was subjected to preparative HPLC separation (system C) and lyophilisation to give a white powder.

^{1}H NMR (400 MHz, [D$_6$]DMSO) δ (ppm): 12.89 – 11.27 (br, 4H), 8.54 (d, J = 7.2 Hz, 1H), 8.26 (d, J = 8.1 Hz, 1H), 8.01-7.89 (m, 3H), 7.50 (d, J = 7.6 Hz, 1H), 5.19 (dd, J = 14.9, 7.6 Hz, 1H), 4.83 (d, J = 13.6, 7.4 Hz, 1H), 4.36 – 4.12 (m, 4H), 4.01 – 3.94 (m, 1H), 3.81 – 3.35 (m, 5H), 2.68 (dd, J = 16.6, 5.5 Hz, 1H), 2.42 (dd, J = 16.6, 8.2 Hz, 1H), 2.37 – 2.07 (m, 4H), 2.08 –

1.62 (m, 13H), 1.55 (ddd, *J* = 19.7, 14.9, 6.5 Hz, 1H), 1.03 (d, *J* = 6.1 Hz, 3H), 0.88 (dd, *J* = 14.9, 6.5 Hz, 6H). ^{13}C NMR (125 MHz, [D$_6$]DMSO) δ (ppm): 174.0, 173.9, 171.8, 171.6, 171.2, 171.1, 170.0, 169.5, 168.9, 168.7, 66.6, 59.5, 58.2, 52.1, 51.6, 47.4, 46.8, 42.0, 41.6, 36.0, 30.2, 30.0, 29.0, 27.2, 26.5, 24.3, 24.0, 22.5, 22.2, 21.5, 19.3. The product exists as a (major/minor 3:1) mixture of rotamers. HRMS (ESI): calculated for $C_{33}H_{51}N_{11}O_{14}$ $[M+H]^+$ 826.3695, found 826.3714. LC-MS: *m/z* (ESI)= 824.55 $[M-H]^-$, t_R= 3.47 min, purity: >95%. HPLC retention time: (system C): t_R= 37.07 min; (system A): t_R= 7.48 min. Purity: >95%. Mass of crude product: 150 mg. Pure peptide obtained from 100 mg of crude: 24 mg.

Compound 9: Ac-Asp-Pro-Glu-Thr-Gly-Glu-Tle-OH

2-Chlorotrityl chloride resin (300 mg, 1.70 mmol/g) was used to prepare this compound according to the general methods. The crude material was subjected to preparative HPLC separation (system D) and lyophilisation to give a white powder.

^{1}H NMR (500 MHz, [D$_6$]DMSO) δ (ppm): 12.76 – 11.55 (br, 4H^r), 8.28 (d, *J* = 8.0 Hz, 1H), 8.06 (dd, *J* = 5.7, 5.6 Hz, 1H), 8.02 – 7.90 (m, 3H), 7.50 (d, *J* = 8.0 Hz, 1H), 4.83 (dd, *J* = 13.6, 8.0 Hz, 1H), 4.45 (dd, *J* = 13.6, 8.2 Hz, 1H), 4.33 – 4.13 (m, 3H), 4.04 (d, *J* = 8.7 Hz, 1H), 3.97 (dt, *J* = 10.5, 6.3 Hz, 1H), 3.93 – 3.56 (m, 5H), 2.68 (dd, *J* = 16.5, 5.5 Hz, 1H), 2.42 (dd, *J* = 16.5, 8.2 Hz, 1H), 2.37 – 2.14 (m, 4H), 2.09 – 1.65 (m, 11H), 1.03 (d, *J* = 6.3 Hz, 3H), 0.94 (s, 9H). ^{13}C NMR (125 MHz, [D$_6$]DMSO) δ (ppm): 174.1, 172.2, 171.8, 171.6, 171.2, 171.1, 170.1, 169.5, 168.9, 168.7, 66.6, 60.4, 59.5, 58.2, 54.9, 52.1, 51.4, 47.4, 46.8, 42.0, 36.0, 33.2, 30.2, 30.1, 29.1, 27.6, 26.6, 24.3, 22.2, 19.4. The product exists as a (major/minor 3:1) mixture of rotamers. HRMS (ESI): calculated for $C_{33}H_{51}N_7O_{16}$ $[M+H]^+$ 802.3470, found 802.3475. LC-MS: *m/z* (ESI)= 800.05 $[M-H]^-$, t_R= 3.35 min, purity: >95%. HPLC retention time (system D): t_R= 38.60 min; (system A): t_R= 7.76 min, purity: >95%. Mass of crude product: 153 mg. Pure peptide obtained from 100 mg of crude: 26 mg.

Compound 10: Ac-Asp-Pro-Glu-Thr-Gly-Glu-Thi-OH

2-Chlorotrityl chloride resin (350 mg, 1.70 mmol/g) was used to prepare this compound according to the general methods. The crude material was subjected to preparative HPLC separation (system E) and lyophilisation to give a white powder.

^{1}H NMR (400 MHz, [D$_6$]DMSO) δ (ppm): 12.85 – 11.50 (br, 4H), 8.31 – 8.20 (m, 2H), 8.05 – 7.88 (m, 3H), 7.49 (d, *J* = 8.0 Hz, 1H), 7.32 (dd, *J* = 5.0, 1.2 Hz, 1H), 6.95 – 6.89 (m, 2H), 4.83 (dd, *J* = 13.7, 8.0 Hz, 1H), 4.45 – 4.12 (m, 6H), 3.99 (dt, *J* = 9.0, 5.8 Hz, 1H), 3.84 – 3.49 (m, 5H), 3.28 (dd, *J* = 14.9, 4.8 Hz, 1H), 3.14 (dd, *J* = 14.9, 8.1 Hz, 1H), 2.68 (dd, *J* = 16.5, 5.5 Hz, 1H), 2.42 (dd, *J* = 16.5, 8.1 Hz, 1H), 2.37 – 2.13 (m, 4H), 2.10 – 1.62 (m, 11H), 1.03 (d, *J*

= 5.8 Hz, 3H). ^{13}C NMR (125 MHz, [D$_6$]DMSO) δ (ppm): 174.1, 174.0, 172.1, 171.8, 171.6, 171.2, 171.1, 170.0, 169.5, 168.9, 168.6, 139.0, 126.8, 126.4, 124.6, 66.6, 59.5, 58.2, 53.5, 52.1, 51.4, 47.4, 46.8, 42.0, 36.0, 30.8, 30.7, 30.2, 29.9, 29.0, 27.5, 26.6, 24.3, 22.2, 19.3. The product exists as a (major/minor 3:1) mixture of rotamers. HRMS (ESI): calculated for $C_{34}H_{47}N_7O_{16}S$ [M+H]$^+$ 842.2878, found 842.2841. LC-MS: *m/z* (ESI)= 840.65 [M−H]$^-$, t_R= 3.43 min, purity: >95%. HPLC retention time (system E): t_R= 46.04 min; (system A): t_R= 7.55 min, purity: >95%. Mass of crude product: 147 mg. Pure peptide obtained from 100 mg of crude: 21 mg.

Compound 11: Ac-Asp-Pro-Glu-Thr-Gly-Glu-Cha-OH

2-Chlorotrityl chloride resin (300 mg, 1.70 mmol/g) was used to prepare this compound according to the general methods. The crude material was subjected to preparative HPLC separation (system F) and lyophilisation to give a white powder.

^1H NMR (400 MHz, [D$_6$]DMSO) δ (ppm): 12.86 – 11.75 (br, 4H), 8.25 (d, J = 7.8 Hz, 1H), 8.13 (d, J = 7.9 Hz, 1H), 8.03 (t, J = 5.7 Hz, 1H), 7.97 (d, J = 8.0 Hz, 1H), 7.89 (d, J = 8.1 Hz, 1H), 7.49 (d, J = 7.9 Hz, 1H), 4.83 (dd, J = 13.7, 8.1 Hz, 1H), 4.45 – 4.12 (m, 5H), 4.04 – 3.93 (m, 1H), 3.82 – 3.34 (m, 5H), 2.68 (dd, J = 16.6, 5.5 Hz, 1H), 2.42 (dd, J = 16.6, 8.1 Hz, 1H), 2.34 – 2.19 (m, 4H), 2.10 – 0.75 (m, 27H). ^{13}C NMR (125 MHz, [D$_6$]DMSO) δ (ppm): 174.1, 174.0, 173.9, 171.8, 171.5, 171.2, 170.9, 170.2, 169.4, 168.9, 168.1, 66.8, 59.4, 58.2, 52.1, 51.5, 49.4, 47.3, 46.7, 42.2, 38.1, 35.8, 33.8, 33.7, 31.0, 30.1, 29.8, 29.0, 27.6, 26.5, 25.9, 25.6, 25.4, 24.8, 22.0, 19.3. The product exists as a (major/minor 3:1) mixture of rotamers. HRMS (ESI): calculated for $C_{36}H_{55}N_7O_{16}$ [M+H]$^+$ 842.3784, found 842.3810. LC-MS: *m/z* (ESI)= 840.85 [M−H]$^-$, t_R= 4.12 min, purity: >95%. HPLC retention time (system F): t_R= 38.60 min; (system A): t_R= 10.30 min, purity: >95%. Mass of crude product: 197 mg. Pure peptide obtained from 100 mg of crude: 16 mg.

Compound 12: Ac-Asp-Pro-Glu-hPhe-Gly-Glu-Leu-OH

Fmoc-Leu-Wang resin (500 mg, 0.69 mmol/g) was used to prepare this compound according to the general methods. The crude material was subjected to preparative HPLC separation (system G) and lyophilisation to give a white powder.

^1H NMR (400 MHz, [D$_6$]DMSO) δ (ppm): 12.78 – 11.62 (br, 4H), 8.27 (d, J = 8.0 Hz, 1H), 8.14 (d, J = 7.7 Hz, 1H), 8.06 (dd, J = 5.8, 5.7 Hz, 1H), 7.91 – 7.77 (m, 3H), 7.31 – 7.21 (m, 2H), 7.21 – 7.13 (m, 3H), 4.84 (dd, J = 14.0, 7.9 Hz, 1H), 4.37 – 4.10 (m, 5H), 3.87 – 3.56 (m, 4H), 2.73 – 2.19 (m, 8H), 2.10 – 1.42 (m, 16H), 0.84 (dd, J = 22.2, 6.5 Hz, 6H). ^{13}C NMR (125 MHz,

[D_6]DMSO) δ (ppm): 174.0, 173.8, 171.9, 171.6, 171.5, 171.1, 171.0, 169.6, 168.9, 168.3, 141.3, 128.3, 128.2, 125.7, 59.7, 52.2, 51.3, 50.2, 47.4, 46.8, 41.8, 36.0, 33.6, 31.1, 30.2, 29.8, 29.0, 27.7, 26.6, 24.2, 24.1, 22.8, 22.2, 21.2. The product exists as a (major/minor 3:1) mixture of rotamers. HRMS (ESI): calculated for $C_{39}H_{55}N_7O_{15}$ [M−H]$^-$ 860.3678, found 860.3631. LC-MS: *m/z* (ESI)= 860.90 [M−H]$^-$, t_R= 4.38 min, purity: >95%. HPLC retention time (system G): t_R= 35.81 min; (system A): t_R= 11.59 min, purity: >95%. Mass of crude product: 167 mg. Pure peptide obtained from 100 mg of crude: 26 mg.

Compound 13: Ac-Asp-Pro-Glu-Bap-Gly-Glu-Leu-OH

Fmoc-Leu-Wang resin (500 mg, 0.69 mmol/g) was used to prepare the resin bound precursor Ac-Asp(OtBu)-Pro-Glu(OtBu)-Dap(Trt)-Gly-Glu(OtBu)-Leu-Wang resin according to the general methods. The resin was treated with TFA/TIS/DCM (5 mL, 3:5:92 *v/v/v*) for 5 min, washed with DCM (3 x 5 mL) and the procedure was repeated twice. A solution of benzoyl chloride (100 µL, 0.863 mmol) and pyridine (140 µL, 1.76 mmol) in DCM (5 mL) was added and the resin was agitated for 5 h. The resin was washed with DCM (3 x 5 mL), DMF (3 x 5 mL) and MeOH (3 x 5 mL) and dried overnight in vacuo. The peptide was cleaved from the resin with Reagent K. The crude material was subjected to preparative HPLC separation (system H) and lyophilisation to give a white powder.

^{1}H NMR (500 MHz, [D_6]DMSO) δ (ppm): 12.63 – 11.88 (br, 4H), 8.46 – 8.36 (m, 1H), 8.31 – 8.14 (m, 3H), 8.06 – 7.98 (m, 2H), 7.90 – 7.78 (m, 3H), 4.82 (dd, J = 13.7, 7.9 Hz, 1H), 4.42 – 4.10 (m, 5H), 3.87 – 3.52 (m, 6H), 2.66 (dd, J = 16.5, 5.5 Hz, 1H), 2.42 (dd, J = 16.5, 7.9 Hz, 1H), 2.37 – 2.09 (m, 4H), 2.07 – 1.42 (m, 14H), 0.84 (dd, J = 29.1, 6.4 Hz, 6H). ^{13}C NMR (125 MHz, [D_6]DMSO) δ (ppm): 174.0, 173.9, 171.9, 171.7, 171.2, 171.0, 170.1, 169.7, 168.9, 168.6, 166.7, 134.2, 131.1, 128.2, 127.3, 59.6, 53.0, 52.3, 51.6, 50.3, 47.5, 46.8, 42.1, 40.8, 36.1, 30.3, 29.9, 29.0, 27.1, 26.7, 24.3, 24.2, 22.5, 22.2, 21.3. The product exists as a (major/minor 3:1) mixture of rotamers. HRMS (ESI): calculated for $C_{39}H_{54}N_8O_{16}$ [M+H]$^+$ 891.3736, found 891.3773. LC-MS: *m/z* (ESI)= 889.65 [M−H]$^-$, t_R= 4.05 min, purity: >95%. HPLC retention time (system H): t_R= 37.01 min; (system A): t_R= 9.58 min, purity: >95%. Mass of crude product: 137 mg. Pure peptide obtained from 100 mg of crude: 27 mg.

Compound 14: Ac-Asp-Pro-Glu-Asn-Gly-Glu-Leu-OH

2-Chlorotrityl chloride resin (500 mg, 1.42 mmol/g) was used to prepare this compound according to the general methods. The crude material was subjected to preparative HPLC separation (system I) and lyophilisation to give a white powder.

^{1}H NMR (500 MHz, [D$_6$]DMSO) δ (ppm): 12.99 – 11.74 (br, 4H), 8.27 (d, J = 7.9 Hz, 1H), 8.14 – 8.00 (m, 2H), 7.99 – 7.80 (m, 3H), 7.48 – 7.40 (br, 1H), 6.99 – 6.91 (br, 1H), 4.83 (dd, J = 13.7, 7.9 Hz, 1H), 4.47 (dd, J = 13.8, 6.8 Hz, 1H), 4.34 – 4.25 (m, 2H), 4.20 – 4.12 (m, 2H), 4.08 – 3.29 (m, 4H), 2.66 (dd, J = 16.4, 5.3 Hz, 1H), 2.59 – 2.46 (m, 2H), 2.42 (dd, J = 16.4, 8.1 Hz, 1H), 2.37 – 2.15 (m, 4H), 2.11 – 1.42 (m, 14H). ^{13}C NMR (125 MHz, [D$_6$]DMSO) δ (ppm): 174.1, 173.1, 171.9, 171.7, 171.6, 171.2, 171.1, 171.0, 169.6, 168.9, 168.4, 59.6, 52.1, 51.5, 50.2, 49.7, 47.5, 46.8, 42.2, 36.9, 36.0, 30.1, 30.0, 29.0, 27.4, 26.7, 24.4, 24.2, 22.7, 22.2, 21.3. The product exists as a (major/minor 3:1) mixture of rotamers. HRMS (ESI): calculated for $C_{33}H_{50}N_8O_{16}$ [M−H]$^-$ 813.3267, found 813.3274. LC-MS: m/z (ESI)= 813.70 [M−H]$^-$, t_R= 3.32 min, purity: >95%. HPLC retention time (system I): t_R= 37.16 min; (system A): t_R= 7.77 min, purity: >95%. Mass of crude product: 119 mg. Pure peptide obtained from 100 mg of crude: 14 mg.

Compound 15: Ac-Asp-Thp-Glu-Thr-Gly-Glu-Leu-OH

2-Chlorotrityl chloride resin (500 mg, 1.42 mmol/g) was used to prepare this compound according to the general methods. The crude material was subjected to preparative HPLC separation (system K) and lyophilisation to give a white powder.

^{1}H NMR (500 MHz, [D$_6$]DMSO) δ (ppm): 12.53 – 11.06 (br, 4H), 8.38 (d, J = 8.0 Hz, 1H^r), 8.15 (d, J = 7.9 Hz, 1H), 8.05 – 7.89 (m, 3H), 7.52 (d, J = 8.3 Hz, 1H), 5.01 (d, J = 8.4 Hz, 1H), 4.90 (dd, J = 16.7, 6.4 Hz, 1H), 4.75 (m, 1H), 4.64 (d, J = 8.4 Hz, 1H), 4.37 – 4.13 (m, 4H), 4.00 – 3.95 (m, 1H), 3.75 (dd, J = 9.8, 5.5 Hz, 2H), 3.31 – 3.23 (m, 2H), 3.06 (dd, J = 12.5, 5.1 Hz, 1H), 2.75 (dd, J = 16.7, 6.1 Hz, 1H), 2.54 – 2.46 (m, 1H) 2.26 (m, 4H), 2.02 – 1.45 (m, 10H), 1.04 (d, J = 6.3 Hz, 3H), 0.86 (dd, J = 23.0, 6.5 Hz, 6H). ^{13}C NMR (125 MHz, [D$_6$]DMSO) δ (ppm): 174.1, 174.0, 173.9, 171.8, 171.1, 171.0, 169.9, 169.3, 169.2, 169.1, 168.5, 158.2, 157.9, 66.6, 62.2, 58.2, 52.2, 51.4, 50.2, 48.9, 47.4, 42.0, 36.0, 32.9, 30.1, 29.9, 27.7, 26.7, 24.2, 22.8, 22.1, 21.3, 19.4. The product exists as a (major/minor 3:1) mixture of rotamers. HRMS (ESI): calculated for $C_{32}H_{49}N_7O_{16}S$ [M+H]$^+$ 820.3035, found 820.3044. LC-MS: m/z (ESI)= 817.95 [M−H]$^-$, t_R= 3.65 min, purity: >95%. HPLC retention time (system K): t_R= 30.14 min; (system A): t_R= 6.30 min, purity: >95%. Mass of crude product: 137 mg. Pure peptide obtained from 100 mg of crude: 12 mg.

Compound 16: Ac-Asp-Pip-Glu-Thr-Gly-Glu-Leu-OH

2-Chlorotrityl chloride resin (250 mg, 1.42 mmol/g) was used to prepare this compound according to the general methods. The crude material was subjected to preparative HPLC separation (system K) and lyophilisation to give a white powder. Mass of crude product: 70 mg. Pure peptide obtained from 70 mg of crude: 3 mg.

HRMS (ESI): calculated for $C_{34}H_{53}N_7O_{16}$ $[M{-}H]^-$ 814.3470, found 814.3454. LC-MS: *m/z* (ESI)= 814.10 $[M{-}H]^-$, t_R= 3.73 min, purity: >95%. HPLC retention time (system K): t_R= 32.36 min; (system A): t_R= 7.40 min, purity: >95%.

Compound 19: (*S*)-(9*H*-Fluoren-9-yl)methyl(1-amino-4-methyl-1-oxopentan-2-yl)-carbamate

The title compound was prepared using an adaptation of a published procedure for Cbz-Leu-NH_2.[1] To a 250 mL round bottomed flask Fmoc-Leu-OH **18** (8.0 g, 22.64 mmol), Boc_2O (6.5 g, 29.61 mmol), $(NH_4)_2CO_3$ (1.4 g, 13.93 mmol) and dry MeCN (90 mL) were added. The reaction mixture was cooled in an ice bath, flushed with Ar and pyridine (2.4 mL, 27.86 mmol) was added dropwise. The ice bath was removed and the reaction was stirred for 16 h at RT. After the addition of H_2O (100 mL), the crude product precipitated as a white solid, which was filtered, washed with H_2O and ice-cold Et_2O and dried in vacuo to give Fmoc-Leu-NH_2 **19** (6.0 g, 84%) as a white powder.

^{1}H NMR (400 MHz, [D$_6$]DMSO) δ (ppm): 7.89 (dd, *J* = 7.6 Hz, 2H), 7.71 (dd, *J* = 7.5, 1.1 Hz, 2H), 7.43 – 7.35 (m, 3H), 7.32 (tdd, *J* = 7.5, 3.6, 1.1 Hz, 2H), 7.28 (br, 1H), 6.95 (br, 1H), 4.36 – 4.15 (m, 3H), 3.95 (ddd, *J* = 10.2, 8.6, 4.9 Hz, 1H), 1.66 – 1.54 (m, 1H), 1.52 – 1.27 (m, 2H), 0.86 (dd, *J* = 14.4, 6.6 Hz, 6H). ^{13}C NMR (125 MHz, [D$_6$]DMSO) δ (ppm): 174.4, 155.9, 143.9, 143.7, 140.7, 127.6, 127.1, 127.0, 125.4, 125.3, 120.1, 65.4, 52.9, 46.7, 40.7, 24.2, 23.0, 21.3. LC-MS: *m/z* (ESI)= 353.15 $[M{+}H]^+$, t_R= 4.54 min, purity: >95%.

Compound 20: (*S*)-(9*H*-Fluoren-9-yl)methyl(1-cyano-3-methylbutyl)carbamate

The title compound was prepared using an adaptation of a published procedure for Cbz-Leu-CN.[1] To a dry 250 mL round bottomed flask were added Fmoc-Leu-NH_2 **19** (3.0 g, 8.51 mmol) and dry DMF (25 mL). The reaction mixture was chilled in an ice bath, flushed with Ar and cyanuric chloride (1.0 g, 5.53 mmol) was added. The ice bath was removed and the reaction was stirred for 16 h at RT. The reaction was quenched with H_2O (50 mL) and extracted with EtOAc (3 x 40 mL). The organic layer was washed with H_2O (3 x 70 mL), dried over anhydrous $MgSO_4$ and evaporated to dryness. The crude solid was dissolved in EtOAc and passed through a short plug of silica. The solvent was removed in vacuo to yield the *α*-amino nitrile **20** (2.8 g, 98%) as a white powder.

^{1}H NMR (500 MHz, [D$_6$]DMSO) δ (ppm): 8.15 (d, *J* = 7.9 Hz, 1H), 7.89 (d, *J* = 7.5 Hz, 2H), 7.69 (d, *J* = 7.5 Hz, 2H), 7.42 (dd, *J* = 7.4, 7.2 Hz, 2H), 7.33 (td, *J* = 7.4, 1.0 Hz, 2H), 4.54 – 4.34 (m, 3H), 4.25 (dd, *J* = 6.6, 6.4 Hz, 1H), 1.69 – 1.57 (m, 3H), 0.87 (dd, *J* = 10.0, 5.0 Hz, 6H). ^{13}C NMR (125 MHz, [D$_6$]DMSO) δ (ppm): 155.4, 143.7, 143.6, 140.7, 127.6, 127.0, 125.1,

125.0, 120.1, 119.8, 65.8, 46.6, 40.6, 24.1, 21.9, 21.4. LC-MS: *m/z* (ESI)= 335.15 [M+H]$^+$, t_R= 7.99 min, purity: >95%.

Compound 21: (*S*)-(9*H*-Fluoren-9-yl)methyl(3-methyl-1-(1*H*-tetrazol-5-yl)butyl)carbamate

The title compound was prepared using an adaptation of a published procedure.[2] To a 100 mL round bottomed flask were added the *α*-amino nitrile **20** (2.0 g, 5.98 mmol), NaN_3 (780 mg, 12 mmol), $ZnBr_2$ (660 mg, 2.93 mmol), iPrOH (9 mL) and H_2O (18 mL). The reaction mixture was stirred at reflux for 16 h. 3 N HCl (5 mL) and EtOAc (30 mL) were added and stirring was continued until no solid was present. The organic layer was isolated and the aqueous layer extracted with EtOAc (2 x 40 mL). The combined organic portions were washed with H_2O (3 x 80 mL), dried over anhydrous $MgSO_4$ and evaporated to dryness. The crude product was crystallised from EtOAc/hexane to yield the *α*-amino tetrazole **21** (1.5 g, 67%) as white crystals.

^{1}H NMR (500 MHz, [D$_6$]DMSO) *δ* (ppm): 13.52 – 12.98 (br, 1H), 8.04 (d, *J* = 8.2 Hz, 1H, CON*H*), 7.89 (d, *J* = 7.5 Hz, 2H), 7.70 (dd, *J* = 8.57, 8.34 Hz, 2H), 7.41 (dd, *J* = 7.4, 7.3 Hz, 2H), 7.32 (dt, *J* = 11.3, 5.8 Hz, 2H), 4.95 (ddd, *J* = 14.6, 8.2, 5.9 Hz, 1H), 4.38 (dd, *J* = 10.2, 7.1 Hz, 1H), 4.29 (dd, *J* = 10.2, 6.9 Hz, 1H), 4.22 (dd, *J* = 6.9, 6.8 Hz, 1H), 1.87 – 1.51 (m, 3H), 0.89 (dd, *J* = 9.3, 6.6 Hz, 6H). ^{13}C NMR (125 MHz, [D$_6$]DMSO) *δ* (ppm): 155.8, 143.8, 143.6, 140.7, 127.6, 127.0, 125.1, 125.0, 120.1, 120.0, 65.6, 46.7, 44.2, 41.4, 24.1, 22.5, 21.4. LC-MS: *m/z* (ESI)= 753.00 [2M−H]$^-$, t_R= 6.56 min, purity: >95%.

Compound 23: (*S*)-2-((((9*H*-Fluoren-9-yl)methoxy)carbonyl)amino)-3-aminopropanoic acid[3]

The title compound was prepared using an adaptation of the method reported for Fmoc-D-Dap-OH.[4] Fmoc-Asn-OH **22** (11.0 g, 31.07 mmol) was added to a solution of (*bis*(trifluoroacetoxy)iodo)benzene (20.2 g, 47.00 mmol) in DMF/H_2O (220 mL, 2:1 *v/v*). After 15 min, pyridine (5.3 mL, 65.53 mmol) was added dropwise, and the mixture was stirred at RT overnight. The solvent was removed under reduced pressure, and the oily residue was re-dissolved in 1N HCl (50 mL). The aqueous solution was washed with Et_2O (4 x 50 mL) and adjusted to pH 6 with 2N NaOH. The resulting precipitate was filtered, washed with H_2O (5 x 30 mL), ice-cold EtOH (10 mL), and ether (3 x 20 mL) and dried in vacuo to give Fmoc-Dap-OH **23** (8.7 g, 86%) as a beige powder.

^{1}H NMR (400 MHz, [D$_6$]DMSO) *δ* (ppm): 9.17 – 7.98 (br, 3H), 7.89 (d, *J* = 7.4 Hz, 2H), 7.70 (d, *J* = 6.8 Hz, 2H), 7.42 (dd, *J* = 7.4, 7.3 Hz, 2H), 7.36 – 7.30 (m, 2H), 6.81 (d, *J* = 6.5 Hz,

1H), 4.35 – 4.20 (m, 3H), 3.71 (m, 1H), 3.00 (dd, *J* = 11.8, 5.0 Hz, 1H), 2.80 – 2.74 (m, 1H). ^{13}C NMR (125 MHz, [D$_6$]DMSO) *δ* (ppm): 171.0, 155.6, 143.8, 143.8, 142.5, 140.6, 139.3, 137.3, 128.9, 127.6, 127.2, 127.0, 125.3, 125.2, 121.3, 120.1, 120.0, 109.7, 65.6, 51.3, 46.5, 40.4.

Compound 24: (*S*)-2-((((9*H*-Fluoren-9-yl)methoxy)carbonyl)amino)-3-(tritylamino)-propanoic acid

The title compound was prepared using an adaptation of a reported procedure.[5] Trimethylsilyl chloride (64 µL, 0.51 mmol) was added to a stirred suspension of Fmoc-Dap-OH **23** (163 mg, 0.51 mmol) in DCM (18 mL) and the mixture was heated at reflux for 2 h and then cooled to RT. Pyridine (140 µL, 1 mmol) and a solution of trityl chloride (140 mg, 0.5 mmol) in DCM (5 mL) was then slowly added and the mixture stirred at RT for a further 1 h. The reaction was quenched with MeOH (10 mL), concentrated in vacuo and the crude product purified by flash chromatography (DCM/hexane 20:80 to 100:0, *v/v*) to afford Fmoc-Dap(Trt)-OH **24** (217 mg, 77%) as a white powder.

^{1}H NMR (500 MHz, [D$_6$]DMSO) *δ* (ppm): 7.89 (d, *J* = 7.6 Hz, 2H), 7.70 (dd, *J* = 7.0, 1.0 Hz, 2H), 7.56 – 7.47 (br, 1H), 7.44 – 7.36 (m, 8H), 7.34 – 7.28 (m, 8H), 7.21 – 7.16 (m, 3H), 4.35 – 4.16 (m, 3H), 4.11 (dd, *J* = 6.4, 6.0 Hz), 2.38 (dd, *J* = 11.6, 6.4 Hz*),* 2.26 (dd, *J* = 11.6, 6.0 Hz, 1H). ^{13}C NMR (125 MHz, [D$_6$]DMSO) *δ* (ppm): 173.1, 155.9, 145.7, 143.7, 140.6, 128.3, 127.7, 127.6, 127.0, 126.1, 125.2, 125.1, 120.1, 70.0, 65.5, 54.7, 46.6, 44.9. LC-MS: *m/z* (ESI)= 1134.35 [2M−H]$^-$, t_R= 7.75 min, purity: >95%.

Fluorescence polarisation assays

The FP assays were carried out as previously described.[6] Varying concentrations of the peptide inhibitor dissolved in DMSO were plated onto untreated Corning® black 96 well plates containing a solution of the Keap1 Kelch domain (200 nM) and the fluorescent peptide FITC-β-DEETGEF-OH (1 nM) in Dulbecco's Phosphate Buffered Saline (DPBS) at pH 7.4 (11% final DMSO concentration, 100 µL final volume). Following 30 min incubation under slow agitation and away from light at RT, the plates were transferred to a PHERAstar microplate reader and the FP was recorded. All measurements were recorded in triplicate. The data were normalised to control and then fitted to a standard four-parameter logistic function using the Origin Pro software.

Differential scanning fluorimetry

The DSF assays were conducted using an adaptation of a published method.[7] Briefly, a solution of the Keap1 Kelch domain protein (5 µM) and detection dye SYPRO® orange (5x) in

DPBS at pH 7.4 was added to the wells of a MicroAmp® Optical 96-well reaction plate containing the peptide inhibitor (10 µM, 10% final DMSO concentration, 40 µL final volume). The plate was sealed using an optical adhesive cover and covered with aluminium foil to protect the dye from light. The plate was then transferred to a plate centrifuge and spun down briefly (200 *g*, 1 min, RT) to remove any bubbles and collect the solution at the bottom of the wells. Following a 30 min incubation at RT, the plate was placed into a 7500 Real time PCR machine and subjected to a standard protein melting protocol[8] using the 7500 Software v.2.0.0. The fluorescence intensity at an excitation of 465 nm and emission of 580 nm was recorded during a temperature scan from 25°C to 95°C with a temperature ramping rate of 1°C/min. All measurements were run in triplicate. The raw data were exported to MS Excel and analysis was performed using a custom script provided by the Structural Genomics Consortium Oxford.[8] The temperature range over which the protein unfolding occurred was selected and the maximum and minimum (at temperatures below the maximum intensity) fluorescence intensities within this range were determined. The processed data were fitted to the Boltzmann equation by linear regression analysis using the Origin Pro software.

Subcloning, expression and purification of Keap1 Kelch protein

The coding sequence for the Keap1 Kelch domain was subcloned into a pEt15b vector as previously described.[9] The plasmids were transformed into *E. coli* BL21 CodonPlus (Novagen) and grown in 6 L of TB (Terrific Broth) at 37°C, supplemented with 100 mg/L ampicillin, to an A_{600} of 1.0 and induced for 24 h with 0.5 mM IPTG (isopropyl β-D-thio-galactoside; Melford) at 20°C. Cell harvesting and subsequent protein purification were performed as previously described with slight modifications.[9] First affinity chromatography was performed using a 5 mL His-Trap column (GE Healthcare, UK) with buffer containing 50 mM HEPES, pH 7.5, 300 mM NaCl and imidazole at 10 mM (lysis buffer), 30 mM (wash buffer) & 250 mM (elution buffer). The purified protein was pooled and passed through a 16/60 Superdex 200 pg size exclusion column with a buffer containing 50 mM HEPES, pH 7.5, 200 mM NaCl to further purify Keap1. The protein was then pooled and concentrated to 10 mg/mL using an Amicon ultraconcentration device (Millipore), aliquoted, flash-frozen in liquid nitrogen, and stored at −80°C for subsequent use.

Isothermal titration calorimetry experiments

The protein was initially dialysed overnight against buffer containing 25 mM HEPES, pH 7.4 and 200 mM NaCl. The protein was diluted to a concentration of 50 µM prior to ITC. Inhibitors were diluted to an appropriate concentration in dialysis buffer so that the final solution

contained no more than 5% DMSO. To maintain a similar buffer composition for titrations, 5% DMSO was added to the protein solution. All peptides in this study were used at a concentration of 500 µM. There were no detectable solubility issues at these concentrations of the peptides in the dialysis buffer. ITC experiments were performed with a MicroCal PEAQ-ITC instrument (Malvern Instruments, UK). Titrations were carried out at 25°C with a stirring speed of around 750 rpm. In total 30 injections were performed for one protein inhibitor experiment with the first injection of 0.3 µL followed by 29 injections of 1.3 µL and a gap of 120 s between each injection. Data analyses were performed after subtraction of the heats of dilution for each experiment. The thermodynamic parameters N (stoichiometry), K_d (dissociation constant/binding affinity), ΔS (entropy change) and ΔH (enthalpy change) were obtained by using the single-site-binding model of the PEAQ-ITC analysis software (Malvern Instruments, UK). For each experiment, at least two titrations were performed. Titration data were analysed independently, and the obtained values were averaged.

Subcloning, expression and purification of Keap1 Kelch protein for crystallisation

The coding sequence for the Keap1 Kelch domain was subcloned into a pEt15b vector as previously described for the ITC experiments.[9] For crystallisation, the same domain was sub-cloned into a modified pEt15b vector with a His-SUMO tag and Ulp1 cleavage site. The plasmids were transformed into *E. coli* BL21 CodonPlus (Novagen) and grown in 6 L of TB (Terrific Broth) at 37°C, supplemented with 100 mg/L ampicillin, to an A600 of 1.0 and induced for 24 h with 0.5 mM IPTG (Melford) at 20°C. Cell harvesting and subsequent protein purification were performed as previously described with slight modifications.[9] First affinity chromatography was performed using a 5 mL His-Trap column (GE Healthcare, UK) with buffer containing 50 mM HEPES, pH 7.5, 300 mM NaCl and imidazole at 10 mM (lysis buffer), 30 mM (wash buffer) & 250 mM (elution buffer). For crystallisation, the purified SUMO-tagged Keap1 Kelch domain with a was pooled and cleaved overnight with Ulp1 protease supplemented with 3 mM DTT and dialysed simultaneously against buffer containing 50 mM HEPES, pH 7.4, 300 mM NaCl. Thereafter the cleaved protein was passed through the His-Trap column for the second time using the same buffers as before. The cleaved protein without the his-tag and sumo tag was captured in the flow through.

The purified proteins (with and without his-tag) was pooled and passed through a 16/60 Superdex 200 pg size exclusion column with a buffer containing 50 mM HEPES, pH 7.5, 200 mM NaCl to further purify Keap1. The protein was then pooled and concentrated to 10 mg/mL using an Amicon ultraconcentration device (Millipore), aliquoted, flash-frozen in liquid nitrogen, and stored at −80°C for subsequent use.

Crystallisation and structure determination of the Keap1 Kelch domain and peptide complex

Crystals for the wild type Keap1-Kelch domain appeared after 2-3 days in 4.0 M ammonium acetate, 0.1 M sodium acetate trihydrate, pH 4.6 at 4°C. The crystals were soaked with individual peptides at 30 mM for 3 h before the crystals were cryo-protected with 20% *w/v* ethylene glycol in 1.2 fold of the crystallisation solution and flash-frozen in liquid nitrogen.

Diffraction data for individual crystals were collected at beamlines I03 at Diamond Light Source. Data were processed using either XDS[10] or iMosflm.[11] The structure of the complex was solved by molecular replacement using the native Keap1 structure (PDB ID: 3ZGD[12]) as a search model. The crystallographic data collection and refinement statistics details are provided in Table 1. The overall Keap1-peptide complexes are similar to the previously published structure.[12] There are small areas of positive and negative electron density in the overall electron density map that cannot be attributed to any model, which is consistent with previous structures. The underlying small percentage of twinning (3-4%) overall as determined by various data quality assessment software available.

Table 1: Data collection, data processing, structure determination and refinement statistics for the binary Keap1-Kelch-domain-peptide complexes. Statistics for the highest-resolution shell are shown in parentheses.

	Ac-DPETGEL-OH (Peptide **5**)	Ac-DEETGEF-OH (Peptide **1**)
PDB ID	6FMQ	6FMP
Data Collection		
Wavelength (Å)	0.9660	0.9660
Resolution range	50 – 2.1 (2.21 - 2.10)	50 – 2.92 (3.02 – 2.92)
Space group	$P2_12_12_1$	$P2_12_12_1$
Unit cell (*a*, *b*, *c*, α, β, γ)	75.65, 76.13, 211.14, 90, 90, 90	76.40, 76.26, 208.29 90, 90, 90
Total reflections	300962 (45442)	53610 (4641)
Unique reflections	71681 (10313)	26886 (2360)
Multiplicity	4.2 (4.4)	2.0 (2.0)
Completeness (%)	99.6 (99.7)	98.9 (92.3)
Mean $I/\sigma(I)$	11.9 (2.6)	7.8 (2.0)
R_{merge}	60 (50.8)	7.8 (62.5)
CC1/2	0.99 (0.49)	0.99 (0.73)
Refinement		
Wilson *B*-factor (Å^2)	43.16	64.31
Average *B*-factor (Å^2)		
Overall	46.34	58.16
Protein	46.00	58.13
Ligand	45.95	71.50
Solvent	51.41	42.21
R_{work}/ R_{free}	16.54/19.97	18.08/23.54
Rmsd, bonds	0.012	0.015
Rmsd, angles	1.203	1.49
Ramachandran plot		
..Favored (%)	97.86	94.35
..Allowed (%)	2.12	5.12
..Outliers (%)	0.02	0.53

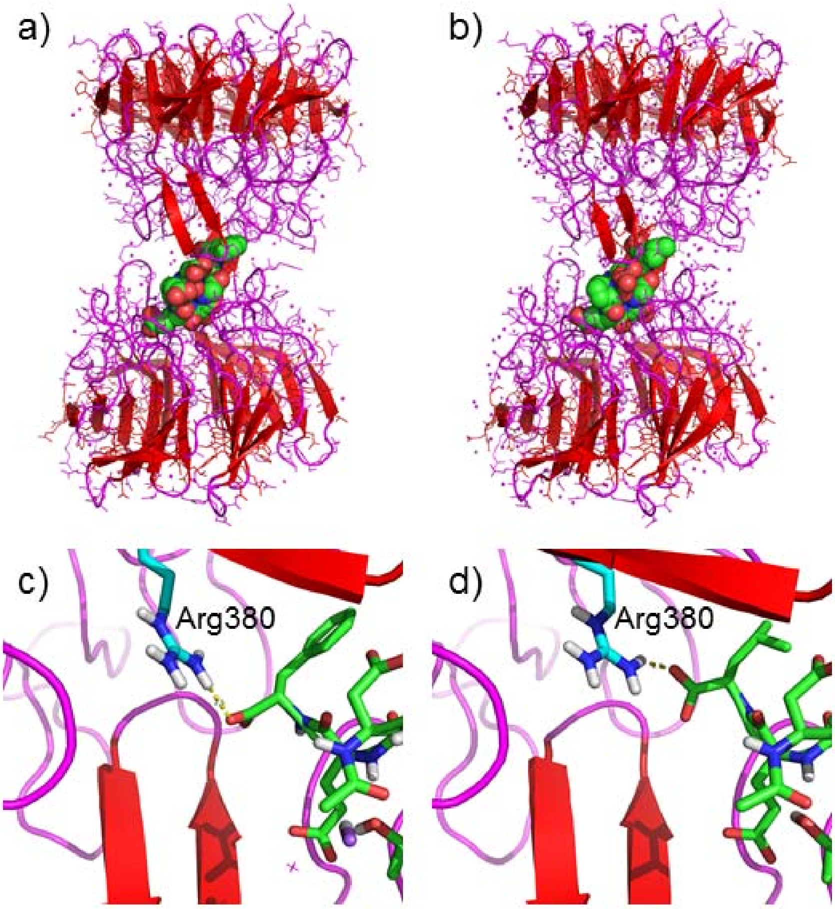

Figure S2. a. and b. Peptide **1** – Keap1 Kelch domain (PDB Ref 6FMP) and peptide **5** – Keap1 Kelch domain (PDB Ref 6FMQ) structures respectively, protein shown in ribbon and stick representation, peptide shown in sphere representation; c. and d. respective interactions between peptides **1** and **5** with the unoccupied Kelch protein showing the interaction between the C-terminal carboxylate of the peptide and Arg380 of the adjacent protein in each structure.

Molecular modelling

Briefly, the modified peptide structures with appropriate side chains were prepared by editing the Keap1 bound conformation of peptide **5** (PDB Ref 6FMQ) using UCSF Chimera 1.12.[13] The images were prepared using Pymol (The PyMOL Molecular Graphics System, Version 1.7.2.1, Schrödinger, LLC).

References

[1] Z. P. Demko, K. B. Sharpless, *Org. Lett.* **2002**, *4*, 2525-2527.

[2] S. Warriner, S. Gunn, A. Baker, R. Bertram, *Synlett* **2007**, *2007*, 2643-2646.

[3] D. Spring, Y. Lau, *Synlett* **2011**, *2011*, 1917-1919.

[4] Y. Rew, M. Goodman, *The Journal of Organic Chemistry* **2002**, *67*, 8820-8826.

[5] F. Wang, J. A. Good, O. Rath, H. Y. Kaan, O. B. Sutcliffe, S. P. Mackay, F. Kozielski, *J. Med. Chem.* **2012**, *55*, 1511-1525.

[6] R. Hancock, H. C. Bertrand, T. Tsujita, S. Naz, A. El-Bakry, J. Laoruchupong, J. D. Hayes, G. Wells, *Free Radic. Biol. Med.* **2012**, *52*, 444-451.

[7] C. Zhuang, S. Narayanapillai, W. Zhang, Y. Y. Sham, C. Xing, *J. Med. Chem.* **2014**, *57*, 1121-1126.

[8] F. H. Niesen, H. Berglund, M. Vedadi, *Nat. Protoc.* **2007**, *2*, 2212-2221.

[9] X. Li, D. Zhang, M. Hannink, L. J. Beamer, *J. Biol. Chem.* **2004**, *279*, 54750-54758.

[10] W. Kabsch, *Acta Crystallogr. D Biol. Crystallogr.* **2010**, *66*, 125-132.

[11] a. T. G. Battye, L. Kontogiannis, O. Johnson, H. R. Powell, A. G. Leslie, *Acta Crystallogr. D Biol. Crystallogr.* **2011**, *67*, 271-281; b. M. D. Winn, C. C. Ballard, K. D. Cowtan, E. J. Dodson, P. Emsley, P. R. Evans, R. M. Keegan, E. B. Krissinel, A. G. Leslie, A. McCoy, S. J. McNicholas, G. N. Murshudov, N. S. Pannu, E. A. Potterton, H. R. Powell, R. J. Read, A. Vagin, K. S. Wilson, *Acta Crystallogr. D Biol. Crystallogr.* **2011**, *67*, 235-242.

[12] S. Horer, D. Reinert, K. Ostmann, Y. Hoevels, H. Nar, *Acta Crystallogr. Sect. F Struct. Biol. Cryst. Commun.* **2013**, *69*, 592-596.

[13] E. F. Pettersen, T. D. Goddard, C. C. Huang, G. S. Couch, D. M. Greenblatt, E. C. Meng, T. E. Ferrin, *J. Comput. Chem.* **2004**, *25*, 1605-1612.

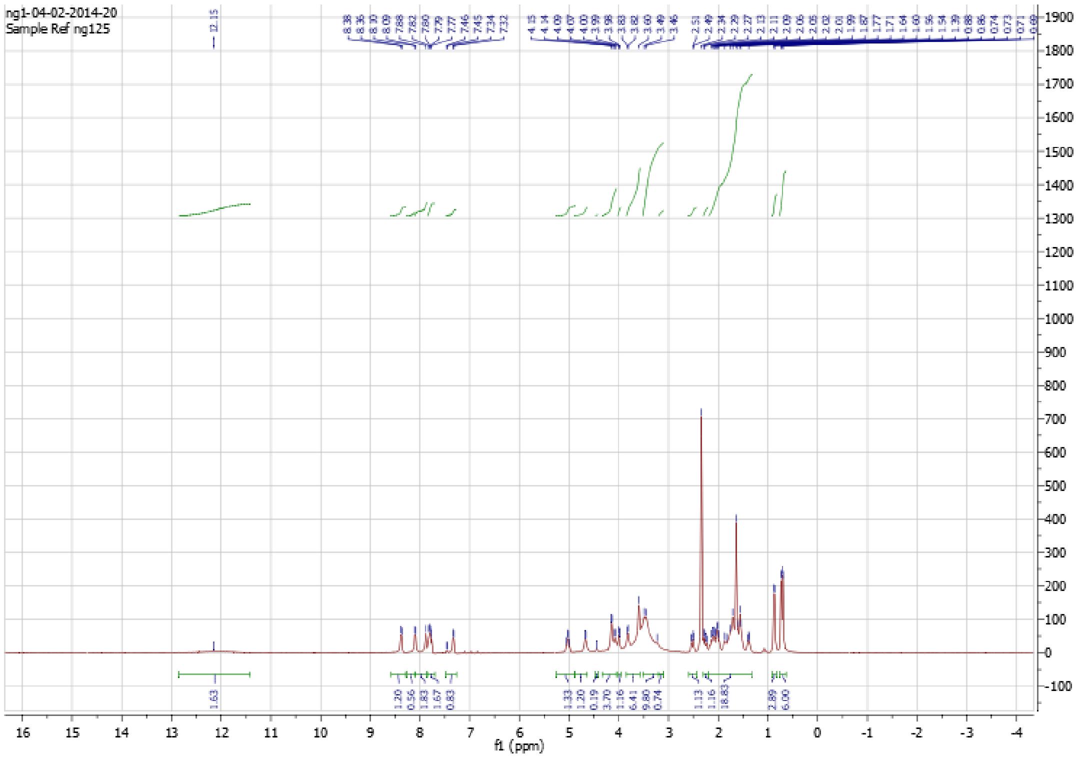

Figure S3. ^{1}H NMR Spectra of compound **8**.

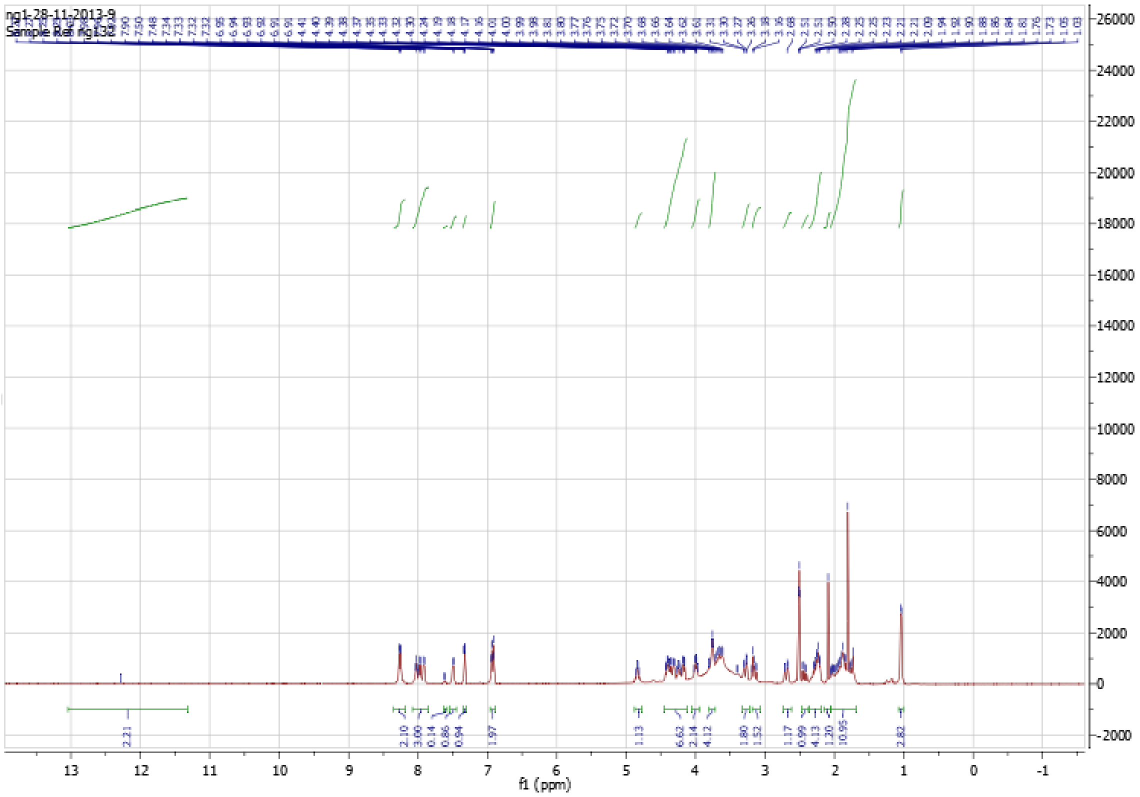

Figure S4. ^{1}H NMR Spectra of compound **10**.

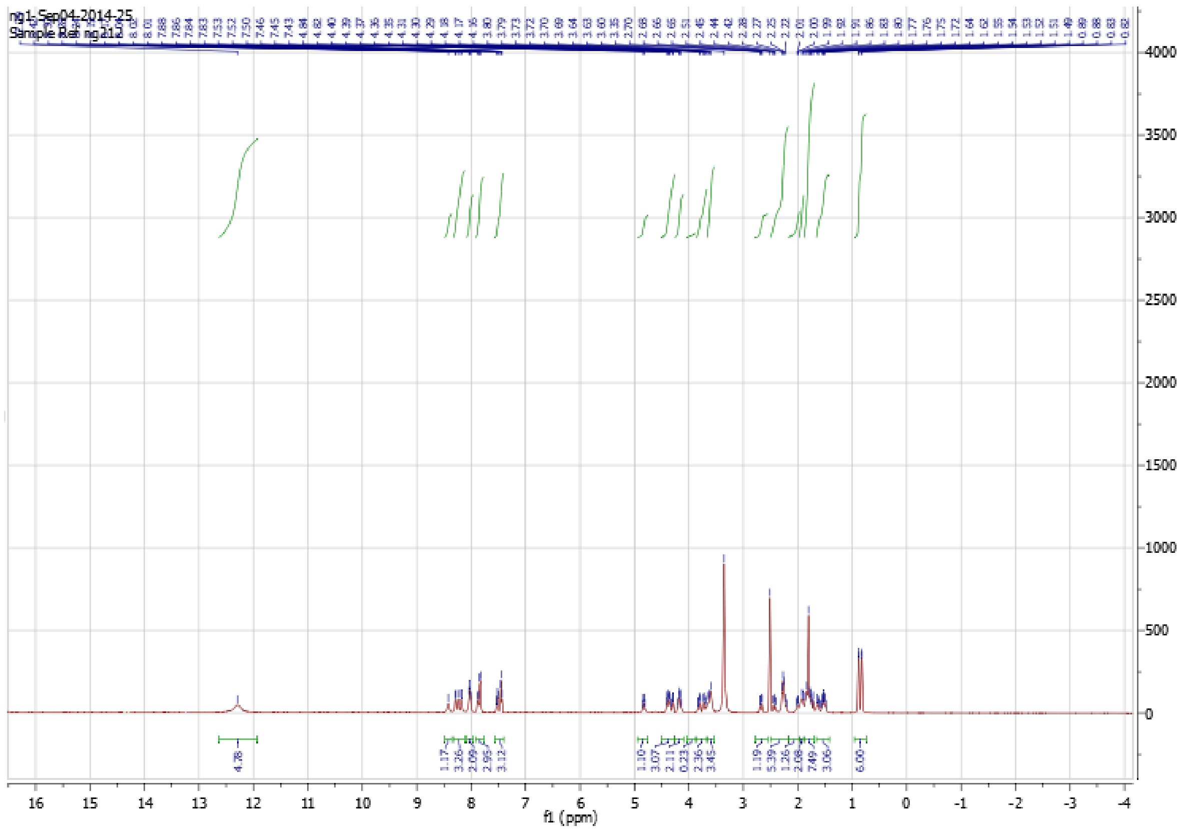

Figure S5. ^{1}H NMR Spectra of compound **13**.

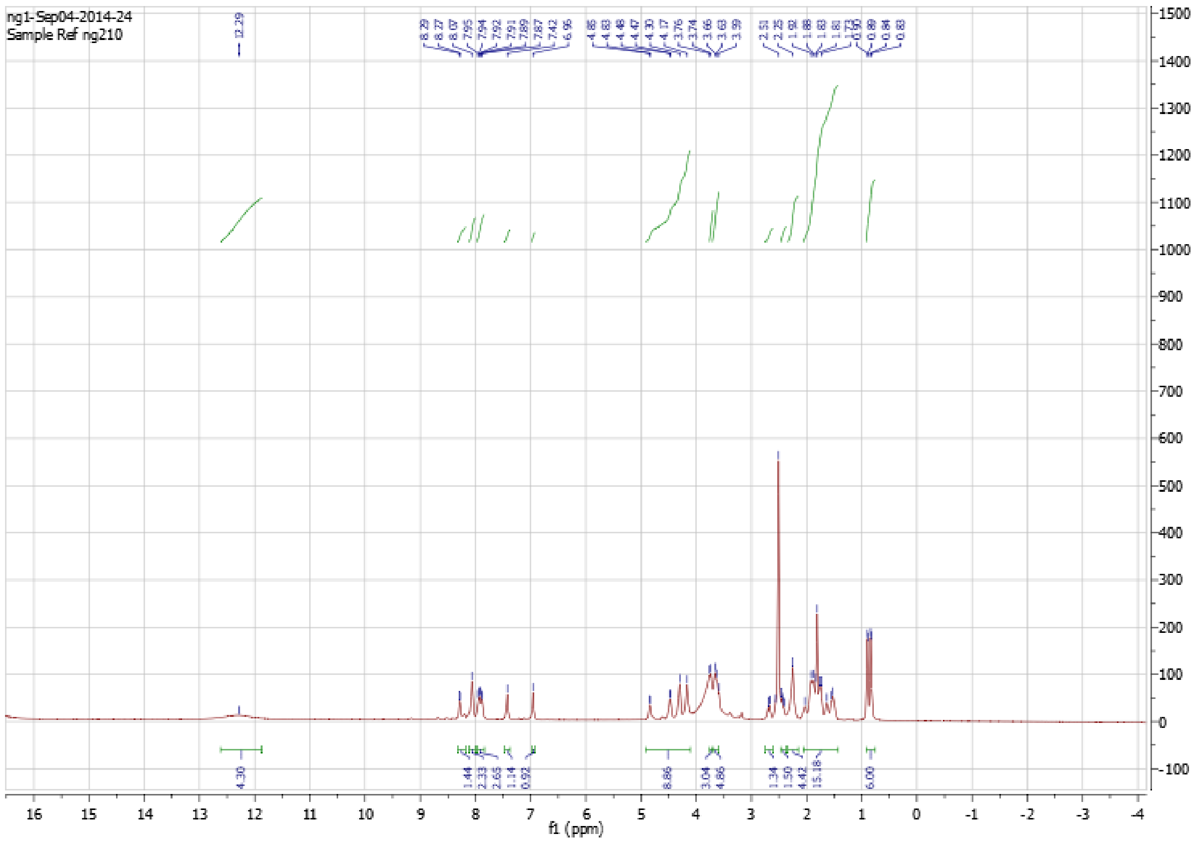

Figure S6. ^{1}H NMR Spectra of compound **14**.

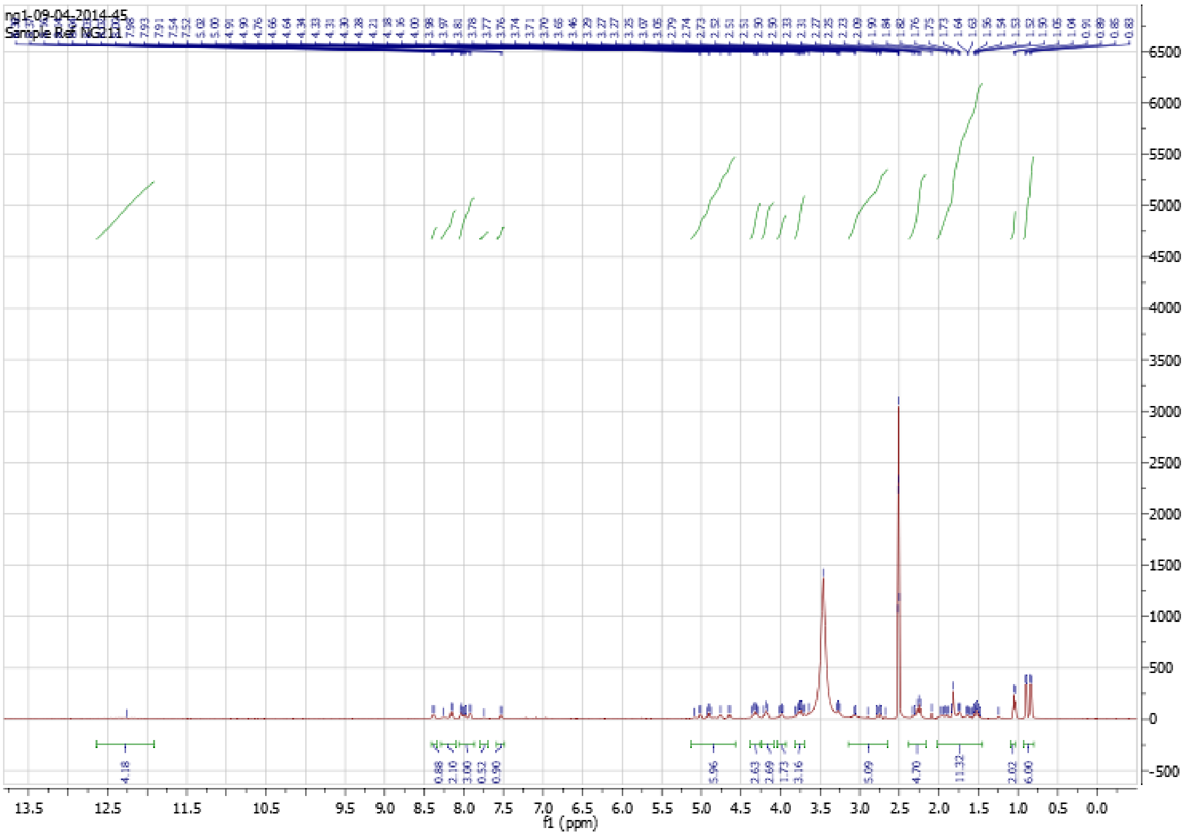

Figure S7. ^{1}H NMR Spectra of compound **15**.

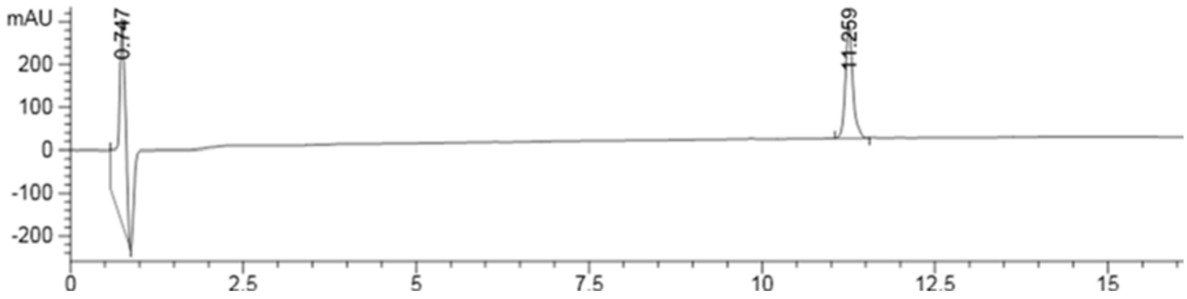

Figure S8. HPLC chromatogram of compound **6**.

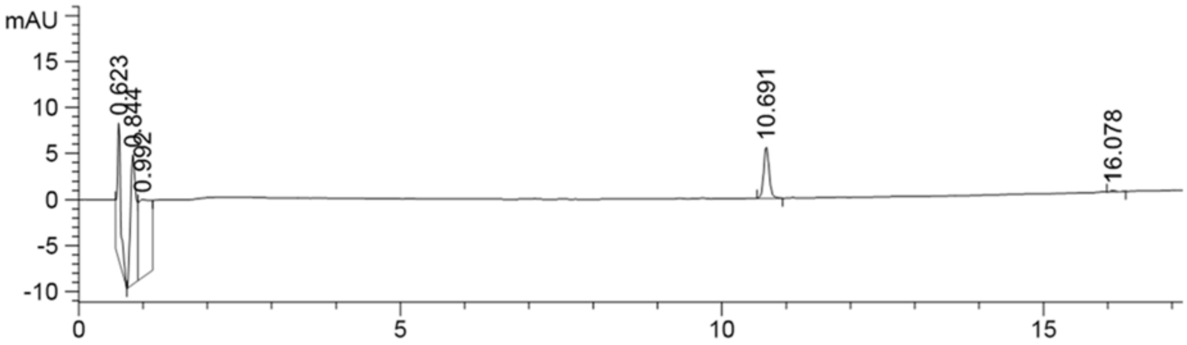

Figure S9. HPLC chromatogram of compound **7**.

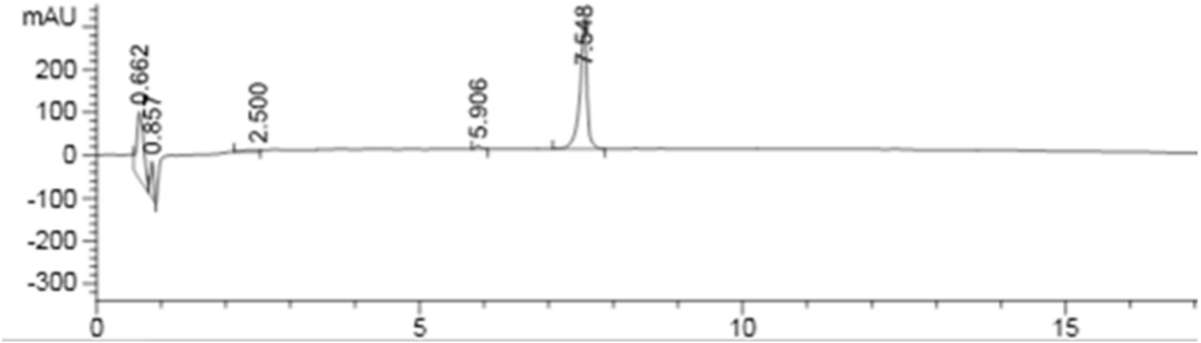

Figure S10. HPLC chromatogram of compound **10**.

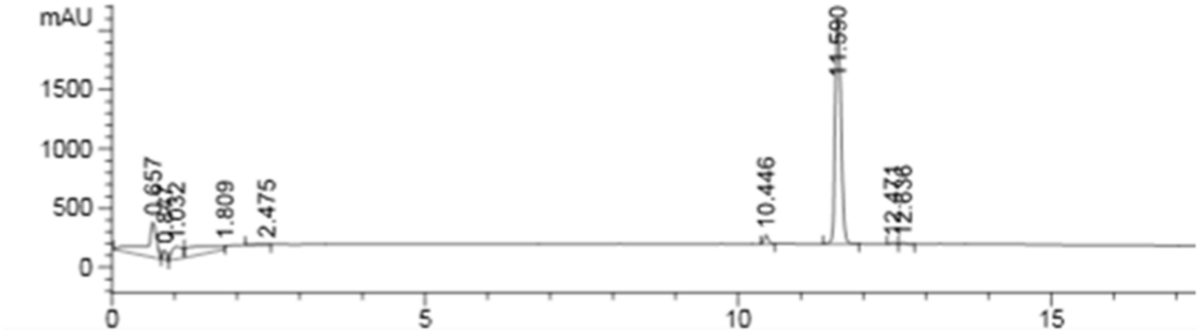

Figure S11. HPLC chromatogram of compound **12**.

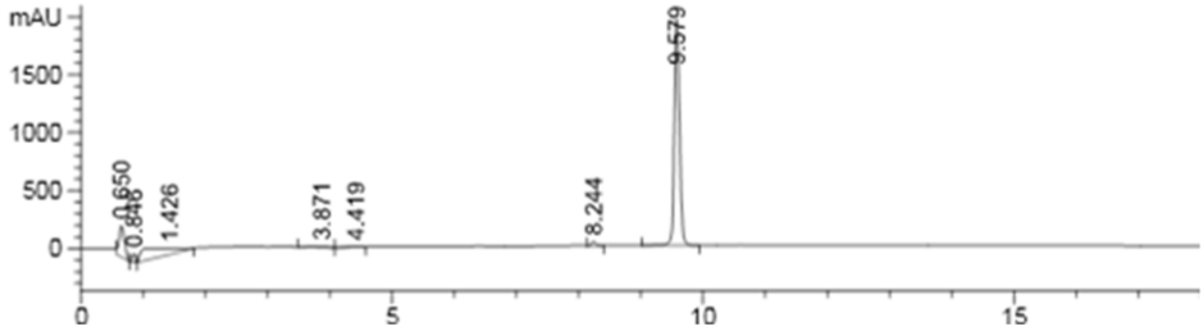

Figure S12. HPLC chromatogram of compound **13**.

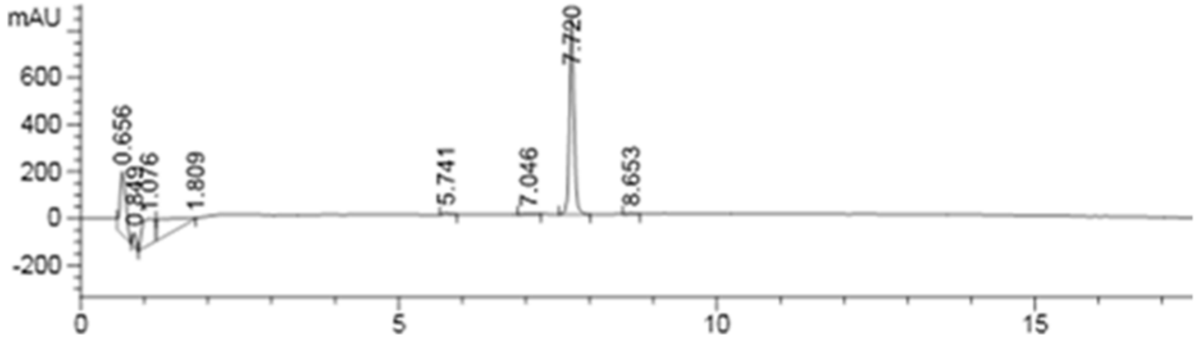

Figure S13. HPLC chromatogram of compound **14**.
